# Decoupling CO_2_ Availability from Bulk Solubility via Biohybrid Reaction–Diffusion Nanoconfinement

**DOI:** 10.1021/jacs.6c12039

**Published:** 2026-08-31

**Authors:** Fhysmélia F. Albuquerque, Artur C. Souza, Matheus S Corsino, Lucas D. Paquini, Graziela C. Sedenho, Rafael N. P. Colombo, Fabio H. B. Lima, Frank N. Crespilho

**Affiliations:** São Carlos Institute of Chemistry, University of São Paulo (USP), 13560-970 São Carlos, Brazil

## Abstract

Although contemporary CO_2_ reduction research is largely dominated by the search for novel active sites, the performance of even the most sophisticated catalysts remains fundamentally constrained by the sluggish mass transport of dissolved CO_2_. Here, we demonstrate that engineering the local chemical microenvironment is as critical as tuning intrinsic catalytic activity. Inspired by the functional orchestration of cyanobacterial carboxysomes, we report a biomimetic interface that integrates carbonic anhydrase within copper phosphate nanoflowers to establish a reaction–diffusion nanoreactor. Hyperspectral micro-FTIR imaging reveals a spatially localized and quantitatively enhanced CO_2_-associated signal at the biohybrid interface, consistent with the formation of an interfacial CO_2_ reservoir sustained by enzymatic bicarbonate dehydration. This interfacial CO_2_ enrichment directly translates into enhanced carbon utilization, manifested by higher conversion rates and improved selectivity toward carbon-derived reduction products relative to the enzyme-free control. Real-time online electrochemical mass spectrometry confirms a shift in selectivity toward carbon-derived products under conditions where conventional electrodes are transport-limited. This work establishes that shifting the focus from material-bound kinetics to interface-driven carbon management is also a strategy for advancing electrosynthesis.

## Graphical abstract

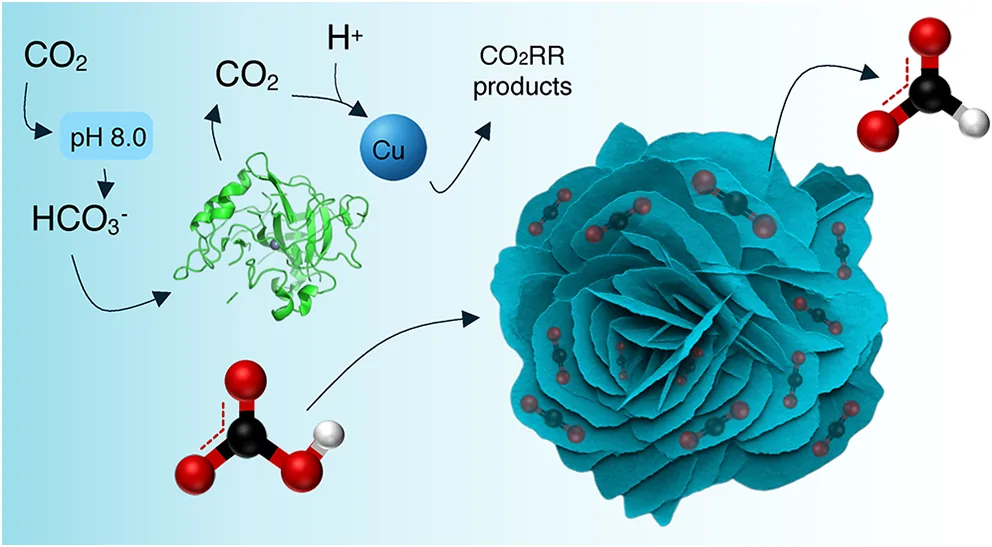

## 1 Introduction

The continuous rise in atmospheric CO_2_ levels has intensified the need for effective decarbonization strategies capable of capturing and transforming this abundant greenhouse gas into value-added products.^1^ In living systems, nature has long mastered carbon management through biological CO_2_ fixation, a process essential for sustaining life on Earth. At the core of this pathway lies ribulose-1,5-bisphosphate carboxylase/oxygenase (RuBisCO), the enzyme responsible for incorporating CO_2_ into organic compounds during the Calvin cycle. However, RuBisCO exhibits low catalytic efficiency, with typical turnover numbers between 2 and 5 s^–1^, and limited specificity for CO_2_ over O_2_, leading to a competing oxygenase reaction known as photorespiration, which reduces photosynthetic efficiency.^2^

To overcome these intrinsic limitations, photosynthetic organisms have evolved CO_2_-concentrating mechanisms (CCMs) that increase the local substrate availability and enhance the carboxylase activity of the enzyme.^3,4^ In cyanobacteria, this process is centered on carboxysomes, protein-based microcompartments that encapsulate RuBisCO together with carbonic anhydrase (CA).^5^ In this system, bicarbonate (HCO_3_^–^) is actively transported and accumulated in the cytosol, then diffuses into the carboxysome, where CA catalyzes its conversion into CO_2_ ^6^ (Figure 1a). The combination of in situ CO_2_ generation and restricted diffusive efflux, possibly imposed by the protein shell, leads to a localized elevation of CO_2_ concentration around the active sites of RuBisCO,^7^ thereby promoting its carboxylase activity and suppressing the competing oxygenase reaction.

**Figure 1:**
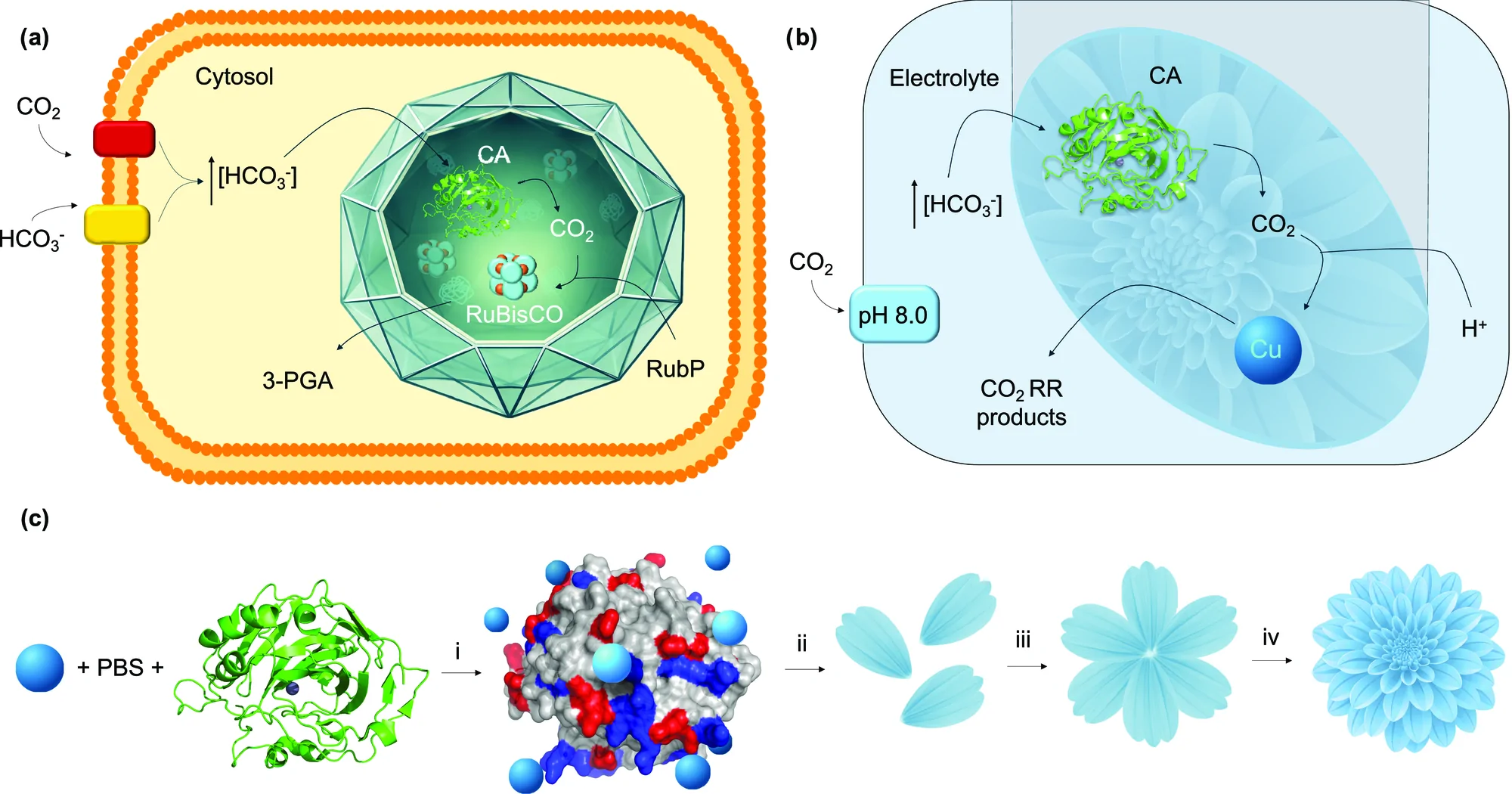
Carboxysome-inspired nanoflowers and their function in local CO_2_ enrichment. (a) Carboxysome model illustrating the CO_2_-concentrating mechanism in cyanobacteria. Inorganic carbon is actively transported across the cell membrane via CO_2_ and HCO_3_^–^ transporters, leading to the accumulation of bicarbonate in the cytosol. HCO_3_^–^ then diffuses into the carboxysome, where carbonic anhydrase (CA) catalyzes its dehydration into CO_2_, resulting in a localized increase in CO_2_ concentration around RuBisCO active sites, thereby enhancing carboxylation and suppressing oxygenation; (b) Biomimetic CA-HNF system mimicking carboxysomal function, in which bicarbonate is converted into CO_2_ in proximity to electrocatalytic sites, enabling localized CO_2_ enrichment and promoting its subsequent conversion into reduction products; and (c) Schematic illustration of the formation of CA-HNF: (i) Crystal structure of carbonic anhydrase (PDB: 1 V9E), which exhibits a net negative surface charge at pH values above 5.1, thereby enabling Cu^2+^ ions (blue spheres) to coordinate with exposed acidic and nitrogen-containing sites, promoting nucleation; (ii) Formation of primary petal-like units; (iii) Self-assembly into larger flower-like structures and (iv) development of the final hierarchical CA-HNF.

Inspired by such natural architectures, artificial systems have sought to mimic the spatial and functional control observed in CCMs to enhance the CO_2_ capture and utilization.^8^ In aqueous media at near neutral or pH > 7, most inorganic carbon exists as bicarbonate rather than dissolved CO_2_. Consequently, the effective concentration of reducible CO_2_ depends on the equilibrium exchange between these two species, which can limit the reaction rate in the absence of efficient CO_2_ regeneration mechanisms.^9^ By mimicking natural CCMs and integrating CA into biomimetic catalysts, CO_2_ can be continuously regenerated in situ, sustaining a local substrate reservoir and thereby enhancing CO_2_ availability for downstream conversion processes.

Importantly, CA does not alter the thermodynamic equilibrium between bicarbonate and CO_2_. Instead, the enzyme accelerates both hydration and dehydration reactions by several orders of magnitude, allowing molecular CO_2_ to be rapidly replenished as it is consumed at nearby catalytic sites. Therefore, local CO_2_ enrichment emerges not from an equilibrium shift, but from the coupling between rapid enzymatic turnover, continuous consumption, and restricted mass transport within confined microenvironments.^4,10^

However, the direct use of enzymes in electrochemical systems is hindered by their fragility under nonphysiological conditions, limited lifetime, and difficulties in recovery and reuse.^11^ To address these challenges, immobilization techniques have been developed to stabilize enzymes while preserving their activity.^12–17^ Among these, the formation of hybrid organic–inorganic nanoflowers (HNFs) offers a compelling strategy.^18–24^ HNFs are hierarchical assemblies typically composed of a protein component and an inorganic phosphate matrix, forming flower-like structures that provide a large surface area, high enzyme loading, and a favorable microenvironment for catalysis. The resulting hybrid materials combine the selectivity of biocatalysts with the robustness of inorganic support. Hybrid nanoflowers are generally employed because their high surface area and bioinorganic organization promote enzyme immobilization, stabilization, and substrate accessibility. In the present system, however, the hierarchical petal-like architecture is hypothesized to provide an additional functional role. The overlapping petals generate interconnected interpetal domains that may increase the effective diffusion-path length and residence time of locally generated molecular species. Accordingly, CA-HNF was selected as a spatially organized reaction–diffusion interface capable of coupling enzymatic bicarbonate dehydration with electrochemical CO_2_ consumption at the adjacent copper-containing phase. Beyond these established advantages, we hypothesized that colocalization of carbonic anhydrase and copper phosphate within a hierarchical nanoflower would enable three complementary functions: rapid regeneration of molecular CO_2_ from bicarbonate, short transport distances between enzymatic generation and electrochemical consumption, and structural modulation of CO_2_ diffusion through the interpetal environment.

Accordingly, despite extensive efforts in catalyst design, limited attention has been given to controlling the local chemical environment at catalytic interfaces; thus, integrating CA within such HNF offers a promising route to design biomimetic catalysts that mirror the cooperative function of carboxysomes. In these architectures, CA can continuously convert bicarbonate into CO_2_ in the confined region of the electrode, while the inorganic matrix drives the subsequent electroreduction of CO_2_ to value-added products.

In this work, we report the development of a biohybrid interface that utilizes a CA-HNF architecture to govern local CO_2_ dynamics (Figure 1b). Spatiotemporal mapping via hyperspectral micro-FTIR reveals a robust CO_2_ enrichment at the interface, driven by enzymatic dehydration of bicarbonate. By decoupling substrate availability from diffusion constraints, this nanoreactor increases local CO_2_ activity, shifting selectivity toward carbon-derived products even under transport-limited conditions. This study establishes interface-mediated carbon flux as a critical lever for optimizing electrochemical CO_2_ reduction, offering a departure from traditional material-centric approaches.

## 2 Materials and Methods

### 2.1 Synthesis and Structural Characterization

Biomimetic CA-HNFs were synthesized via a protein-mediated coprecipitation route. Briefly, 20 μL of an aqueous CuSO_4_ solution (120 mmol L^–1^) was added to 3 mL of PBS buffer (0.1 mol L^–1^, pH 7.4) containing carbonic anhydrase at concentrations ranging from 0 to 0.5 mg mL^–1^. The mixtures were incubated at 25 °C for 72 h without agitation. The resulting blue precipitates were isolated by centrifugation, washed three times with deionized water, and lyophilized. The resulting samples were designated CA-HNF005, CA-HNF01, and CA-HNF05, according to the CA concentration (0.05, 0.1, and 0.5 mg mL^–1^, respectively) used during synthesis. Unless otherwise stated, the designation CA-HNF refers specifically to CA-HNF005 throughout the Results and Discussion sections of this article. The enzyme loading was evaluated by UV–vis spectroscopy (see Supporting Information, Section 3). Morphology was examined by field-emission scanning electron microscopy (FE-SEM, JEOL JSM-7200F) at an accelerating voltage of 5.0 kV, while elemental distributions were confirmed by energy-dispersive X-ray spectroscopy (EDS) mapping. High-resolution X-ray photoelectron spectroscopy (XPS; O 1s, C 1s, P 2p, N 1s and Cu 2p) was employed to probe the surface chemical composition and the electronic environment of the bioinorganic interface. Among these, the Cu 2p environment was monitored before and after electrolysis (see Supporting Information, Section 4).

### 2.2 Electrochemical Performance and Product Analysis

Bioelectrodes were fabricated by dispersing the hybrid catalysts in an agarose/glutaraldehyde (1.5 mg mL^–1^/1.25%) hydrogel matrix, which was drop-cast onto HEDGE graphite substrates and dried under vacuum. HEDGE graphite was selected as the conductive substrate because its high density of exposed edge planes provides favorable electrochemical accessibility and adhesion for agarose-immobilized particulate catalysts. Previous direct comparison using the same hydrogel immobilization strategy demonstrated a higher electrochemical response for HEDGE than for conventional glassy carbon and showed the formation of a homogeneous and stable hydrogel coating on the graphite surface.^25^ Electrochemical CO_2_ reduction was conducted in a conventional three-electrode cell using CO_2_-saturated Tris-HCl buffer (0.1 mol L^–1^, pH 8.0) (see Supporting Information, Section 2, for the full bioelectrode assembly protocol and cell configuration). Catalytic performance was evaluated by cyclic voltammetry (10 mV s^–1^) and 100 h chronoamperometry at −1.1 V vs Ag/AgCl. Aliquots of the liquid-phase products were collected, filtered, and analyzed by high-performance liquid chromatography (HPLC; Shimadzu Prominence UFLC). Samples were injected into a Bio-Rad Aminex HPX-87H column maintained at 40 °C and eluted isocratically with 3.33 mmol L^-1^ H_2_SO_4_ at a flow rate of 0.6 mL min^-1^. The total run time and injection volume were 25 min and 50 μL, respectively. The separated species were detected using a UV detector (Shimadzu SPD-20A) at 210 nm. The gas-phase products were monitored by online electrochemical mass spectrometry (EC-MS) at *m*/*z* = 2 (hydrogen), *m*/*z* = 15 (methane), and *m*/*z* = 28 (carbon monoxide) using a Pfeiffer QMA 200 quadrupole mass spectrometer. Faradaic efficiencies were calculated based on the total charge passed (see Supporting Information, Section 8 for equations).

### 2.3 Hyperspectral Micro-FTIR Imaging and Simulations

The spatiotemporal dynamics of bicarbonate-to-CO_2_ conversion were visualized using hyperspectral micro-FTIR imaging. A Bruker Vertex 70 V spectrometer was coupled to a Hyperion 3000 microscope equipped with a liquid nitrogen-cooled 64 × 64 focal plane array (FPA) detector. This configuration enabled the simultaneous acquisition of 4,096 spatially resolved spectra per scan, providing a diffraction-limited chemical map of the interface. To minimize spectral interference from water, 0.1 M NaHCO_3_ solutions were prepared in D_2_O and drop-cast onto CA-HNF samples deposited on gold substrates. Chemical maps were constructed by integrating the area under the asymmetric CO_2_ stretching band (ν_3_ ≈ 2350 cm^–1^) as well as the protein amide I and II bands. The contribution of atmospheric CO_2_ was removed by subtracting an ambient air background spectrum. These experimental observations were corroborated by numerical simulations designed to reproduce the spatial resolution, focal volume, and field of view of the FPA system. The simulations describe the CA-HNF as a reaction–diffusion nanoreactor in which the enzyme maintains local thermodynamic equilibrium under steady-state conditions (see Supporting Information, Section 6 for full simulation parameters and custom Python scripts).

## 3 Results and Discussion

### 3.1 Protein-Directed Assembly and Interfacial Properties of Biomimetic Nanoreactors

The immobilization of CA in HNF occurs through basically four steps. Figure 1c illustrates this process, highlighting the acidic and basic regions of carbonic anhydrase in red and blue, respectively. Once the synthesis is carried out at a pH above the isoelectric point of CA (5.1), its surface remains predominantly negatively charged. Consequently, copper ions can interact electrostatically with the protein backbone and coordinate to the nitrogen-containing groups^26^ step (i). These initial protein-metal interactions serve as nucleation points that trigger the growth of inorganic–organic petals step (ii). As the process continues, the petals self-assemble into larger, flower-like architectures step (iii), ultimately giving rise to three-dimensional aggregates of overlapping “petals”, typical of self-assembled Cu_3_(PO_4_)_2_-based materials step (iv).^27–29^

The morphology of the samples was examined by FE-SEM. As shown in Figure 2a–l, both nanoflowers synthesized in the absence of the enzyme and those formed in the presence of CA exhibit a characteristic hierarchical flower-like structure. The incorporation of CA during synthesis markedly influenced the structural organization of the nanoflowers. Compared with NF, the CA-containing samples displayed more defined petals, greater uniformity, and enhanced compactness, features indicative of protein-mediated control over nucleation and crystal growth. As the CA concentration increased from 0.05 to 0.5 mg mL^–1^, the petal density progressively increased, leading to more compact and hierarchically organized morphologies (Figure 2e–h). This trend agrees with previous studies showing that higher biomolecule concentrations promote multiple nucleation events by increasing the number of coordination sites available for metal ions.^18,30^ Beyond the overall morphology, closer inspection of the petal surfaces revealed clear textural changes between NF and CA-HNF samples (Figure 2i–l). The NF petals exhibited smooth and homogeneous surfaces, whereas those of CA-HNFs appeared rougher. This alteration in texture suggests the successful incorporation of the enzyme, as the adsorption and coordination of protein molecules at the crystal surface can disrupt the uniform growth of the inorganic matrix, leading to an irregular texture.

**Figure 2:**
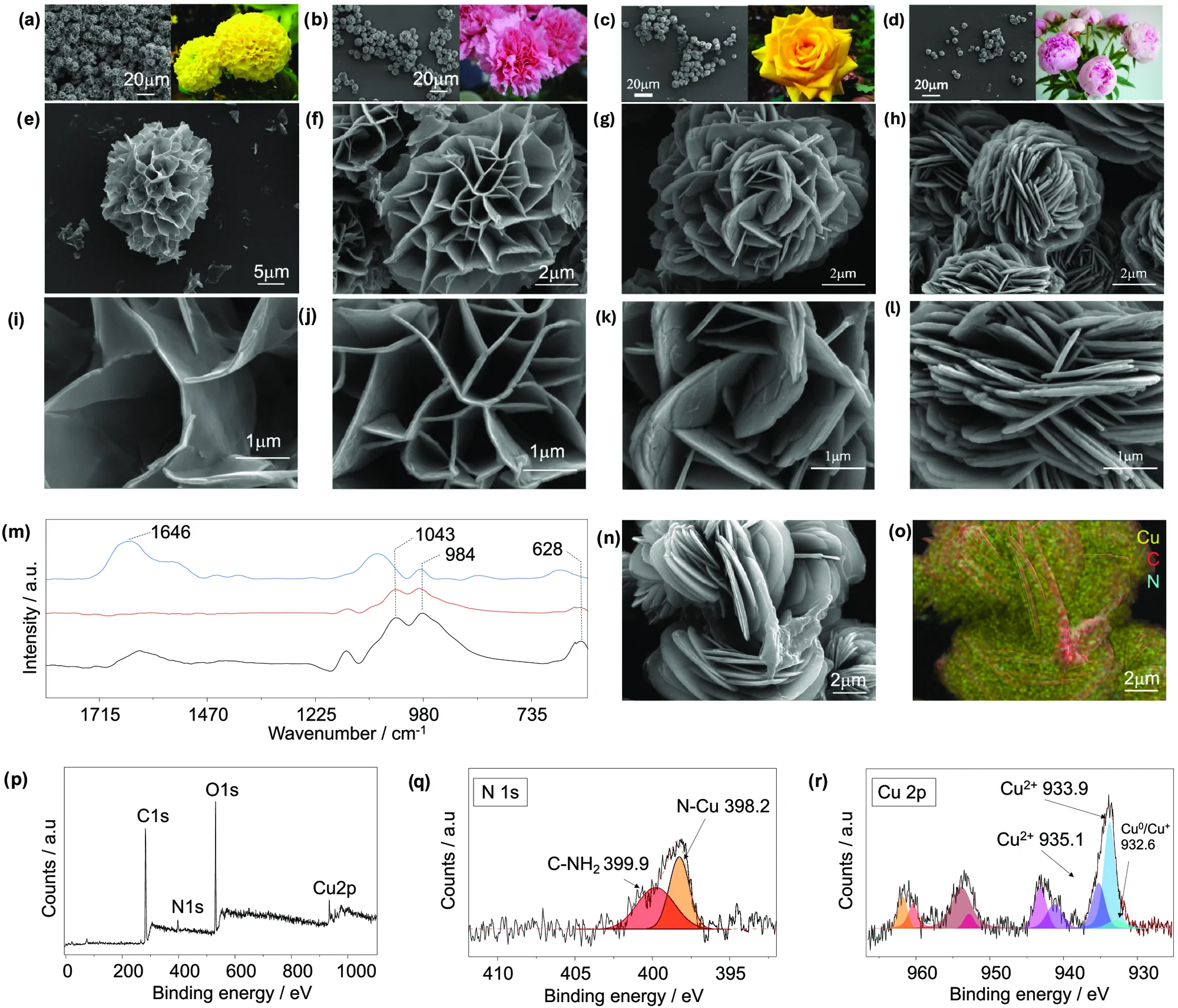
Structural characterization of CA-HNF. (a-l) FE-SEM images of nanoflowers synthesized with increasing CA concentrations: (a, e, i) 0 mg mL^–1^, (b, f, j) 0.05 mg mL^–1^, (c, g, k) 0.1 mg mL^–1^ and (d, h, l) 0.5 mg mL^–1^; (a–d) Low-magnification views illustrating the overall morphology and their visual analogy to ornamental flowers (insets); (e–h) Higher-magnification images of individual nanoflowers highlighting the increase in petal density as CA concentration increases; (i–l) Close-up views of the petal surfaces, showing textural variations associated with enzyme content; (m) FTIR spectra of free CA (blue line), NF (red line), and CA-HNF (black line), evidencing characteristic amide bands from the enzyme and phosphate vibrations from the inorganic matrix; (n, o) EDS mapping confirming the spatial distribution of Cu (yellow), C (red) and N (cyan) within the hybrid structures; (p) Survey CA-HNF XPS spectrum and high resolution spectra in (q) N 1s and (r) Cu 2p regions with raw spectra in solid lines and the cumulative fit peak in dashed lines.

The chemical identity of these hybrids was first confirmed by FTIR analysis (Figure 2m), which revealed characteristic vibrational bands from both the enzyme and the inorganic phosphate matrix. The free CA spectrum displayed amide I and II bands at 1646 and 1543 cm^–1^ associated with peptide backbone vibrations,^31,32^ whereas the NF spectrum showed strong P–O stretching bands at 1043 and 984 cm^–1^ and an O–P–O vibration at 628 cm^–1^, typical of phosphate groups in Cu_3_(PO_4_)_2_ structures.^32^ Minor shifts in the amide region suggest subtle conformational adjustments during assembly.^20^ To further investigate the spatial distribution of these components, elemental mapping by EDS was performed (Figure 2n,o). The composite image in Figure 2o, which provides a spatial overlay of the individual signals shown in Figures S2a–c, confirmed a homogeneous distribution of copper, consistent with the inorganic Cu_3_(PO_4_)_2_ phase.

In contrast, the signals corresponding to carbon and nitrogen, markers of the protein component, were more heterogeneous, supporting the successful incorporation of CA into the hybrid framework.

Following structural analysis, XPS was employed to investigate the surface chemical composition and electronic environments of the elements involved in the CA-HNF structure. The survey spectrum (Figure 2p) confirms the presence of C, O, N, and Cu in the sample, as expected for the CA-HNF. The high-resolution O 1s, C 1s, and P 2p spectra are provided in the Supporting Information (Figure S3). Analysis of the N 1s region (Figure 2q) reveals two main components located at 398.2 and 399.9 eV. The peak at 399.9 eV is attributed to amine and amide nitrogen species (C-NR_2_ and N–C=O),^33^ which are characteristic of peptide bonds and side-chain functionalities of the immobilized enzyme. The lower binding energy component at 398.2 eV is attributed to Cu–N interaction.^34^ In the CA-HNF, this signal may therefore be associated with the interaction of Cu^2+^ species with pyridinic nitrogen from histidine residues of the enzyme. The Cu 2p region (Figure 2r), as expected for Cu_3_(PO_4_)_2_, shows the Cu 2p_3_/_2_ and Cu 2p_1_/_2_ peaks at binding energies of 933.6 and 953.8 eV, respectively, accompanied by characteristic shakeup satellite features.^35^ XPS studies report that Cu^2+^ species interacting with nitrogen-containing ligands exhibit Cu 2p signals in the 935.0–935.4 eV range.^34^ Accordingly, the contribution at 935.1 eV is attributed to Cu^2+^ species interacting with nitrogen functionalities from the immobilized enzyme, in agreement with the N 1s analysis. After electrolysis, XPS analysis reveals a partial reduction of Cu^2+^ species, with an increase in the relative fraction of reduced copper species (Cu^0^/Cu^+^) (Figure S3d). The ex situ XPS results demonstrate cathodically induced restructuring of the copper-containing surface but do not establish a direct, time-resolved relationship among copper oxidation state, catalytic current, and product selectivity. The increased contribution of reduced copper species may be associated with the working electrocatalytic interface. However, the performance of CA-HNF cannot be attributed solely to copper reduction, because the enzyme-free NF and catalytically inactive protein controls contain comparable copper phosphate phases but exhibit substantially lower electrochemical responses.

While FTIR and XPS analyses confirm the physical presence of CA within the nanoflower structure, these spectroscopic signatures alone cannot establish whether the immobilized enzyme retains catalytic function. To directly address this, the enzymatic activity of CA-HNF was evaluated using the Wilbur-Anderson electrometric assay, which monitors the time required for a CO_2_-saturated solution to lower the pH of a Tris-HCl buffer from 8.3 to 6.3. Using equal total masses of material, free CA reduced this time from an uncatalyzed background of 58.1 ± 2.5 s to 21.0 ± 1.6 s, while CA-HNF reduced it to 26.1 ± 6.8 s, corresponding to Wilbur-Anderson units of 1.77 and 1.23, respectively (Figure S4). This indicates that the immobilized CA retains approximately 69% of the catalytic enhancement observed for the free enzyme, despite constituting only a fraction of the total composite mass. These results confirm that the encapsulated CA remains catalytically active within the CA-HNF architecture.

The present XPS measurements do not establish whether partial reduction of the copper phosphate phase alters the molecular activity of immobilized carbonic anhydrase. The Wilbur-Anderson assay confirms that CA retains substantial catalytic activity after immobilization, while the electrochemical stability measurements demonstrate preservation of the overall functional response of the biohybrid electrode during operation. However, an enzyme-specific activity assay performed after electrolysis was not conducted.

### 3.2 Interfacial CO_2_ Concentration at CA-HNF Evidenced by Micro-FTIR Imaging

At pH 8, most dissolved inorganic carbon exists in the form of bicarbonate, whereas the concentration of free CO_2_(aq) is comparatively low. This distribution arises from the acid–base equilibrium shown in eq 1, whose p*K*_a_ (6.35) leads to a [HCO_3_^–^]/[CO_2_] ratio of approximately 45:1.

1 $$CO_{2}\left(g\right)+H_{2}O⇌H^{+}+\mathrm{HC}O_{3}^{-},K_{a}=4.45\times 10^{-7}$$

Consequently, at pH > 6.35, the availability of CO_2_ for reactions is not determined solely by its bulk concentration, but also by the rate at which CO_2_ can be regenerated from bicarbonate near the interfaces.^36^ In this context, the presence of CA, which catalyzes the reversible dehydration of bicarbonate with a *k*_cat_ = 3.5 × 10^5^ s^–1^, is expected to modulate the interfacial CO_2_ concentration.

To directly probe the CCM at the biointerface of CA-HNF, we employed state-of-the-art hyperspectral micro-FTIR imaging equipped with a FPA detector. Unlike conventional point-scan mapping, the FPA architecture functions similarly to a high-resolution digital camera sensor, where each of the 64 × 64 pixels acts as an independent detector. This setup allows for the simultaneous acquisition of thousands of spatially correlated spectra, providing a diffraction-limited chemical map of the interface. This high-throughput spectroscopic approach was critical to visualizing the transient CO_2_ gradients; it enabled us to resolve the chemical landscape of the reaction–diffusion nanoreactor with unprecedented spatiotemporal precision, revealing the spatially localized distribution of the CO_2_-associated absorbance signal, generated by the enzymatic dehydration of bicarbonate, an observation that would be masked by the averaging inherent in bulk or single-point spectroscopic techniques. This approach allows not only the identification of chemical species but also the visualization of their distribution within heterogeneous samples. The hyperspectral maps represent the spatial distribution of the integrated absorbance bands and should therefore be interpreted as relative signal distributions rather than as direct measurements of absolute local concentration. Accordingly, the regions highlighted in the optical images of CA-HNF (Figure 3a) and control NF (Figure 3e) were selected for hyperspectral analysis.

**Figure 3:**
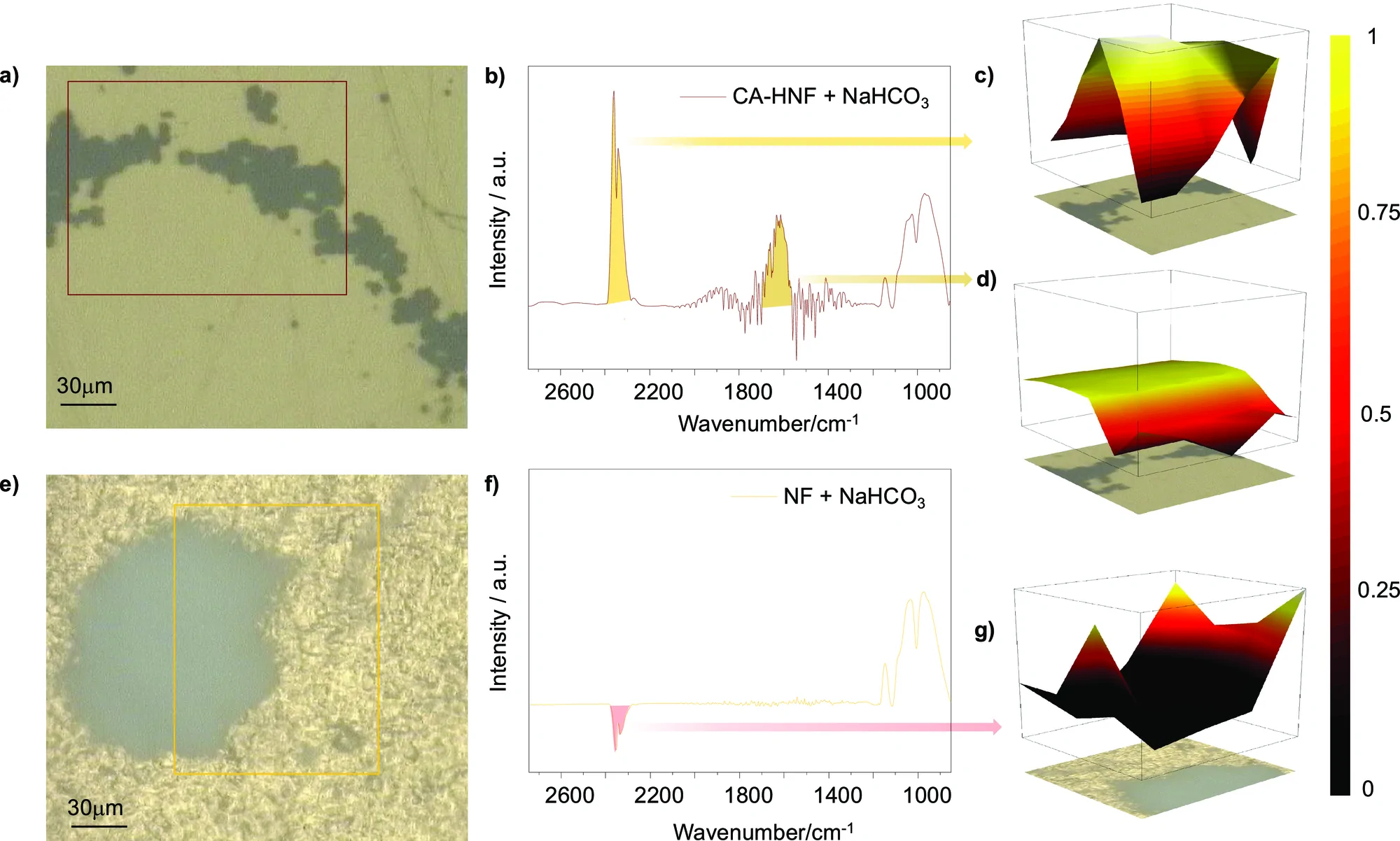
Interfacial CO_2_ accumulation at CA-HNF revealed by spatially resolved micro-FTIR. Optical image of (a) CA-HNF and (e) NF. The black and red squares indicate the regions selected for spectral acquisition and subsequent chemical image construction; FTIR spectra of (b) CA-HNF and (f) NF in the presence of bicarbonate solution, highlighting the vibrational bands integrated to generate the chemical images; Chemical images showing the spatial distribution of (c) 2350 cm^–1^, assigned to O=C=O stretching, and (d) 1600 cm^–1^, assigned to amide absorption, for CA-HNF spectra and (g) 2350 cm^–1^ for NF. All CA-HNF and NF maps are displayed using the same fixed integrated-absorbance color scale, enabling direct visual comparison between the two materials.

The spectra obtained from CA-HNF exposed to the NaHCO_3_ solution (Figure 3b) display the characteristic O=C=O asymmetric stretching band of CO_2_ at approximately 2350 cm^–1^. In contrast, the spectra of NF (Figure 3f) do not show a representative absorbance at this wavenumber, providing direct spectroscopic evidence of interfacial CO_2_ accumulation favored by CA.

To visualize the distribution, chemical maps were constructed by integrating the CO_2_ absorption band (∼2350 cm^–1^). These maps reveal that regions of higher CO_2_ concentration (yellow areas) are spatially confined to zones directly above the particles of CA-HNF samples (Figure 3c). Such spatial heterogeneity is a key strength of FTIR imaging, as it reflects localized chemical environments rather than average bulk properties. This spatial correlation rules out atmospheric CO_2_ as the origin of the observed signal, as a homogeneous contribution from the atmosphere would result in a uniform color distribution across the mapped area.

To further validate the origin of the CO_2_ signal, chemical maps were generated by integrating the amide bands characteristic of proteins (Figure 3d). These maps confirm that enzyme-rich regions are colocalized with the nanoflower structures. Notably, the spatial overlap between the amide and CO_2_ signals provides compelling evidence that the accumulation of CO_2_ originates from the enzymatic conversion of bicarbonate at the CA-HNF interface. This spatial correlation, enabled by hyperspectral imaging, establishes a direct link between enzyme localization and chemical transformation.

For comparison, chemical maps were also constructed by integrating the CO_2_ absorption band for the NF spectrum under identical experimental conditions (Figure 3g). In this case, the resulting map is dominated by blue homogeneous regions, indicating a low and atmospheric CO_2_ concentration across the analyzed area. The absence of a localized CO_2_ signal in the NF control is mechanistically significant: NF possesses the same hierarchical, porous petal architecture as CA-HNF, and is therefore equally capable, in principle, of retaining solution species through geometric confinement alone. If passive physical trapping within the porous framework were sufficient to generate the observed CO_2_ reservoir, a comparable localized signal would be expected for NF as well. Its absence indicates that porosity alone cannot account for the CO_2_ accumulation observed in CA-HNF, and instead points to a coupled mechanism in which enzymatic CO_2_ generation and structural confinement act together, consistent with the spatial overlap between the CO_2_ and protein amide maps.

To evaluate whether the spatial localization observed in D_2_O was preserved in a protic medium, an additional hyperspectral micro-FTIR experiment was performed using bicarbonate prepared in H_2_O (Figure S5). Despite the stronger aqueous background and lower signal-to-noise ratio, the ν_3_(CO_2_) band at approximately 2350 cm^–1^ remained detectable, and the corresponding three-dimensional maps showed localized CO_2_-associated intensity in regions containing CA-HNF. This qualitative agreement indicates that the spatial localization is not produced exclusively by the use of D_2_O.

To corroborate the experimental spatial profiles, we performed hyperspectral micro-FTIR simulations modeling the CA-HNF as a 100-μm irregular particle embedded in a bicarbonate solution. By treating carbonic anhydrase as a kinetic accelerator that maintains local thermodynamic equilibrium, we mapped the steady-state CO_2_ concentration gradients. These concentration fields were converted into normalized absorbance values (*A*/*A*_eq_), bypassing the need for adjustable scaling factors and allowing a direct comparison with experimental data. The high fidelity between the simulated and observed CO_2_ landscapes confirms that the interfacial signal originates from a robust CO_2_ reservoir sustained by the enzymatic interconversion of bicarbonate, rather than optical or geometric artifacts. The full simulation protocol is available in the Supporting Information. While CA does not alter the equilibrium position of the CO_2_/HCO_3_^–^ system, its exceptionally high turnover rate ensures rapid interconversion, sustaining a finite pool of molecular CO_2_ wherever local gradients or confinement arise. Simulated optical image (Figure S6a), two and three-dimensional micro-FTIR maps (Figure S6b,c), constructed on micrometric grids matching the experimental focal volume, reproduce the spatial features observed experimentally. When normalized by the equilibrium absorbance (*A*_2350_/*A*_2350_,eq) and rendered using the same color palette as the experimental data, the simulations show a pronounced enrichment of CO_2_ at the CA-HNF interface, with a steep decay toward bulk solution values. This agreement confirms that the experimental color gradients directly reflect true concentration variations rather than optical artifacts or baseline effects.

The close correspondence between experiment and simulation provides compelling proof that CA-HNFs promote localized accumulation of molecular CO_2_ at the solid–liquid interface. This interfacial enrichment emerges from the interplay of fast enzymatic turnover, thermodynamic equilibrium, and spatial confinement, and explains the intense and localized CO_2_ signatures observed by micro-FTIR chemical maps.

### 3.3 Mass Transport and Confinement in CA-HNF Interfaces

The performance of the CA-HNF system emerges from a tightly coupled interplay between enzymatic kinetics, mass transport, and geometric confinement, placing it in a fundamentally different operational regime compared to conventional electrocatalytic interfaces. A central principle underlying biological CCMs is the preferential accumulation of inorganic carbon in the form of bicarbonate rather than dissolved CO_2_. In classical systems, substrate availability is governed by diffusion from the bulk electrolyte, typically resulting in the formation of a micrometric diffusion boundary layer that limits reaction rates. In contrast, the incorporation of CA within the HNF architecture dynamically reshapes the local chemical environment, giving rise to a confined reaction–diffusion domain in which CO_2_ is continuously generated, retained, and consumed at the interface.

Under the reduced-order steady-state approximation, the local CO_2_ distribution normal to the interface is described by

$$D_{\mathrm{eff}}\frac{d^{2}C}{dx^{2}}-k_{\mathrm{eff}}\left(C-C_{\mathrm{bulk}}\right)=0$$

where *D*_eff_ is an effective diffusion coefficient and *k*_eff_ is a lumped first-order parameter representing the combined effects of accessible enzyme activity, local enzyme concentration, substrate conditions, and internal transport. Given the high catalytic turnover of CA, the enzyme effectively maintains local thermodynamic equilibrium on time scales much shorter than diffusion. As a consequence, the solution to the governing equation adopts an exponential form

$$C\left(x\right)={C_{\mathrm{bulk}}+(C}_{0}-C_{\mathrm{bulk}})e^{-x/\delta }$$

where *C*_0_ is the interfacial CO_2_ concentration and δ is the characteristic diffusion length, defined as

$$\delta =\sqrt{\frac{D_{\mathrm{eff}}}{k_{\mathrm{eff}}}}$$

The parameter δ is a model-derived reaction–diffusion decay length and is not interpreted as a directly measured pore dimension, petal spacing, hydrogel thickness, or electrochemical diffusion-layer thickness.

In the absence of enzymatic activity, considering *K*_eff_ = 3.06 × 10^–2^ s^–1^, the CO_2_ concentration profile is dictated solely by diffusion from the bulk, resulting in a relatively shallow gradient and a diffusion layer thickness on the order of tens of micrometers δ_NF_ ∼ 250 μm. As plotted in Figure 4a, this extensive diffusion layer creates a substrate deficit near the electrode surface, a transport bottleneck that becomes even more evident when observing the flat, multimicrometric concentration plateau in the logarithmic scale of Figure 4b. Under these conditions, the supply of reducible CO_2_ is intrinsically limited by transport across this extended region, leading to inefficient utilization of inorganic carbon species, particularly under near-neutral or basic pH, where bicarbonate dominates. In striking contrast, the presence of CA within the nanoflower architecture fundamentally alters this picture. The experimental results demonstrate localized enhancement of the relative CO_2_-associated signal, while the reduced-order model predicts that enzyme-assisted interconversion may produce a shorter characteristic reaction–diffusion length than in the enzyme-free system. Using an effective reaction rate derived from the reported catalytic turnover frequency of carbonic anhydrase together with the diffusion coefficient of dissolved CO_2_, the characteristic reaction–diffusion length is estimated to be δ_CA_ ∼ 100 nm. Figure 4 illustrates the concentration profiles predicted by the reduced-order reaction–diffusion model for the selected effective parameters, where the macro-scale diffusion layer completely collapses, being replaced by an ultrathin, localized concentration spike confined to the immediate vicinity of the biocatalytic interface. It is important to emphasize that while the normalized profile (*C*/*C*_0_) features a sharper spatial decay for the biohybrid system due to this nanometric confinement, the absolute local concentration of CO_2_ at the immediate electrode surface (*C*/*C*_0_) is drastically higher in the CA-HNF than in the enzyme-free NF under operational conditions. While the conventional NF suffers from severe local substrate depletion driven by slow bulk transport, the CA-HNF acts as a continuous, *in situ* molecular factory that converts the abundant local bicarbonate pool into active CO_2_, thus sustaining a steady-state interfacial substrate activity that is orders of magnitude higher than its bulk solubility limits. The effective first-order rate constant used in the model is based on the rapid catalytic interconversion promoted by carbonic anhydrase and should not be interpreted as a direct operando measurement of immobilized-enzyme kinetics. The Wilbur-Anderson assay confirms that CA-HNF retains substantial catalytic activity after immobilization; however, variations in enzyme accessibility, local pH, confinement, and mass transport may affect the effective kinetic parameter under electrochemical conditions.

**Figure 4:**
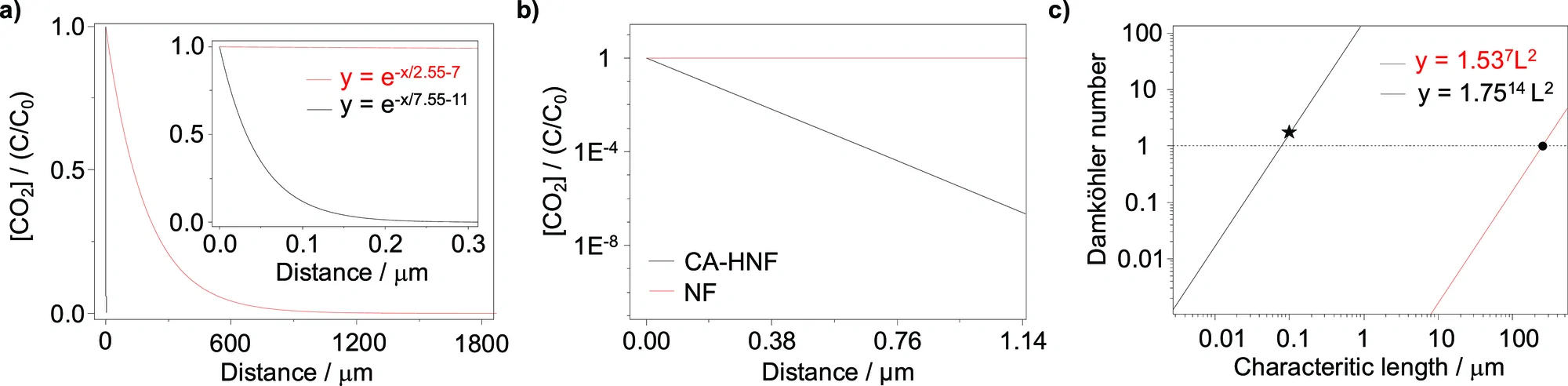
Calculated CO_2_ concentration profiles and Damköhler number analysis. (a) One-dimensional normalized concentration profiles (*C*/*C*_0_) as a function of distance from the interface for the CA-HNF (black line) and enzyme-free NF (red line) systems; the inset shows a magnified view of the near-interface region from 0.0 to 0.3 μm; (b) the corresponding concentration profiles plotted on a logarithmic scale; (c) Damköhler number as a function of the characteristic length scale for the CA-HNF (black line) and enzyme-free NF (red line) systems.

Enzyme immobilization can, in principle, alter catalytic activity relative to the free enzyme, either through conformational restriction or through structural/microenvironmental effects specific to hybrid nanoflowers. To assess this experimentally, the catalytic activity of CA-HNF relative to free CA was evaluated using the Wilbur–Anderson assay, which showed that the immobilized enzyme retains approximately 69% of the catalytic enhancement observed for the free enzyme under matched total sample mass. This indicates that the literature-derived *k* value used in the model may modestly overestimate the effective rate constant of the immobilized CA, and that a corrected, experimentally informed *k* would yield a more conservative estimate of the local CO_2_ enrichment predicted by the model.

To quantitatively assess the relative importance of reaction and transport, the Damköhler number (Da) is introduced as

$$\mathrm{Da}=\frac{k_{\mathrm{eff}}\cdot L^{2}}{D_{\mathrm{eff}}}$$

where *L* is the characteristic length scale of the system. Here, *L* represents an effective characteristic transport length within the heterogeneous nanoflower architecture rather than a directly measured pore or interpetal dimension. Because CA-HNF contains a distribution of interconnected voids, petal separations, and tortuous transport pathways, L should be interpreted as an averaged descriptor of the spatial scale over which reaction and diffusion are coupled. Substituting *k*_cat_ = 3.5 × 10^5^ s^–1^, *L* ∼ 10^–7^ m, and *D* = 2 × 10^–9^ m^2^ s^–1^, yields

Da≈1.75

Because *L* and *k*_eff_ are not independently measured within the hydrated nanoflower, Da is presented as a sensitivity map over the investigated parameter space. The reported Da ≈ 1.75 corresponds to one illustrative parameter combination and is not treated as an experimentally determined material constant. The rate constant *k* was initially estimated from literature turnover values for free CA in solution. To obtain a more conservative, experimentally constrained estimate, *k* was corrected using the activity ratio measured by the Wilbur-Anderson assay, in which CA-HNF retained approximately 69% of the catalytic enhancement observed for free CA. Applying this correction factor decreases the estimated Damköhler number from Da ≈ 1.75 to Da ≈ 1.2, and increases the corresponding characteristic reaction–diffusion length by approximately 20%. This value, modestly above unity (Da > 1), places the system in a reaction–diffusion coupled regime where enzymatic conversion and mass transport operate on comparable time scales, with the reaction rate slightly exceeding the diffusive flux.^37^ This operational state is visually mapped in Figure 4c, which establishes that as long as the nanoreactor operates within these nanometric length scales, the generated CO_2_ is molecularly trapped and preserved inside the porous petals before it can escape into the bulk electrolyte, effectively acting as a localized trapping domain analogous to biological carbon-concentrating mechanisms.

Further insight into the coupled dynamics of the system is obtained by comparing the characteristic time scales of diffusion and reaction. The diffusion time across the confined region can be estimated as

$$\tau_{\mathrm{diff}}=\frac{L^{2}}{D}\approx 5\times 10^{-6}\;s$$

while the enzymatic reaction time is given by

$$\tau_{\mathrm{rxn}}=\frac{1}{k_{\mathrm{cat}}}\approx 3\times 10^{-6}\;s$$

The comparable magnitude of these time scales indicates a tightly coupled reaction–diffusion system operating near the transition between kinetic and transport control. The Damköhler number near unity confirms that the system operates at the interface between kinetic and transport control, with neither process overwhelmingly dominant, a regime that maximizes the coupling efficiency between enzymatic CO_2_ generation and its local consumption.

Beyond enzymatic acceleration, the hierarchical architecture of the nanoflowers introduces an additional level of control through geometric confinement. In a heterogeneous hierarchical architecture, overlapping petals and branching interpetal voids may increase the effective tortuosity of molecular diffusion pathways. An increase in tortuosity reduces the effective diffusivity relative to the unrestricted solution and may consequently increase the residence time of enzymatically generated CO_2_ near the electrochemically active phase. The densely packed petal structures increase the tortuosity of diffusion pathways and reduce the effective diffusivity according to

$$D_{\mathrm{eff}}=\frac{D}{\tau }$$

where τ is the tortuosity factor associated with the porous network. As the density of petals increases, as observed by SEM with increasing enzyme incorporation, the effective diffusivity decreases and the residence time of CO_2_ within the structure correspondingly increases

$$\tau_{\mathrm{res}}∼\frac{L^{2}}{D_{\mathrm{eff}}}$$

This geometric effect reinforces the enzymatic confinement by prolonging the time available for reaction before diffusion into the bulk can occur. The combined effect of reduced diffusivity and high catalytic turnover leads to the accumulation of CO_2_ within the nanoflower structure, as directly visualized by spatially resolved micro-FTIR chemical mapping.

The efficiency of this confinement can be quantified by the ratio between interfacial and bulk CO_2_ concentrations

$$\eta_{\mathrm{conf}}=\frac{C_{\mathrm{interface}}}{C_{\mathrm{bulk}}}$$

Considering bulk CO_2_ concentrations in the 10^–4^–10^–3^ M range and normalized interfacial maxima observed in FTIR maps, the system achieves a confinement efficiency on the order of η_conf_ ∼ 10–10^2^, corresponding to an enhancement in local CO_2_ availability. This amplification is directly correlated with structural features such as petal density and compactness, establishing a clear structure–function relationship between nanoflower morphology and catalytic performance.

These results define a unified physical picture in which the CA-HNF system operates as a reaction–diffusion nanoreactor characterized by (i) the reduced-order reaction–diffusion model predicts that inclusion of enzyme-assisted interconversion decreases the characteristic spatial decay length relative to the enzyme-free case, (ii) operation in a reaction–diffusion coupled Damköhler regime (Da ≈ 1.75), (iii) strong coupling between reaction kinetics and mass transport, and (iv) geometric confinement-induced modulation of diffusivity and residence time. The emergence of a self-sustained interfacial CO_2_ reservoir under these conditions represents a departure from traditional catalyst design paradigms, where active site engineering alone is typically emphasized.

In this context, the CA-HNF architecture demonstrates that controlling the local transport landscape, through the synergistic combination of enzymatic catalysis and hierarchical nanostructuring, can be as critical as tuning intrinsic catalytic activity. By transforming mass transport from a limiting factor into a design parameter, this system establishes molecular confinement and interfacial chemical gradients as central elements.

It should be noted that the reaction–diffusion model presented above is a simplified, one-dimensional representation of a system that is, in reality, a complex three-dimensional hierarchical architecture, with heterogeneous petal density, branching interpetal voids, and a distribution of pore sizes and shapes rather than a single, uniform characteristic length scale. The calculated concentration enhancement, characteristic diffusion length, and Damköhler number are model-derived quantities based on effective kinetic and transport parameters and do not represent direct operando measurements of the interfacial CO_2_ concentration. Because enzyme immobilization may alter the effective catalytic activity, its potential impact on the model was assessed by conservatively scaling the effective rate constant using the experimentally observed CA-HNF/free-CA activity ratio of approximately 0.69. Under this assumption, the Damköhler number would decrease from approximately 1.75 to approximately 1.2, while the characteristic reaction–diffusion length would increase by approximately 20%. These estimates would nevertheless remain consistent with a coupled reaction–diffusion regime in which reaction and transport occur on comparable characteristic time scales.

### 3.4 Biointerfacial Microenvironment Engineering Enhances Carbon Utilization and Electrosynthetic Efficiency

The cyclic voltammograms presented in Figure 5a illustrate the electrochemical behavior of the different materials immobilized on the electrode, with the maximum current density (*J*_max_) at −1.7 V highlighted in Figure 5b. In comparison, the hydrogel alone (background) produced only a negligible current density (−0.63 ± 0.13 mA cm^–2^), confirming its lack of catalytic activity. The enzyme-free NF exhibited a current density of −6.19 ± 0.16 mA cm^–2^, which stems from the intrinsic electrocatalytic activity of copper phosphate toward CO_2_ reduction, while the CA-HNF reached the highest value (−9.34 ± 0.12 mA cm^–2^). As indicated by capacitance analysis in Figure 5c, the improvement in reaction rate between NF and CA-HNF is not favored by the electroactive area, once the *C*_dl_ of NF is higher, but the current reached by CA-HNF is increased by more than 50%. This evidence indicates a significant enhancement in the rate of reduction reactions upon enzyme immobilization.

**Figure 5:**
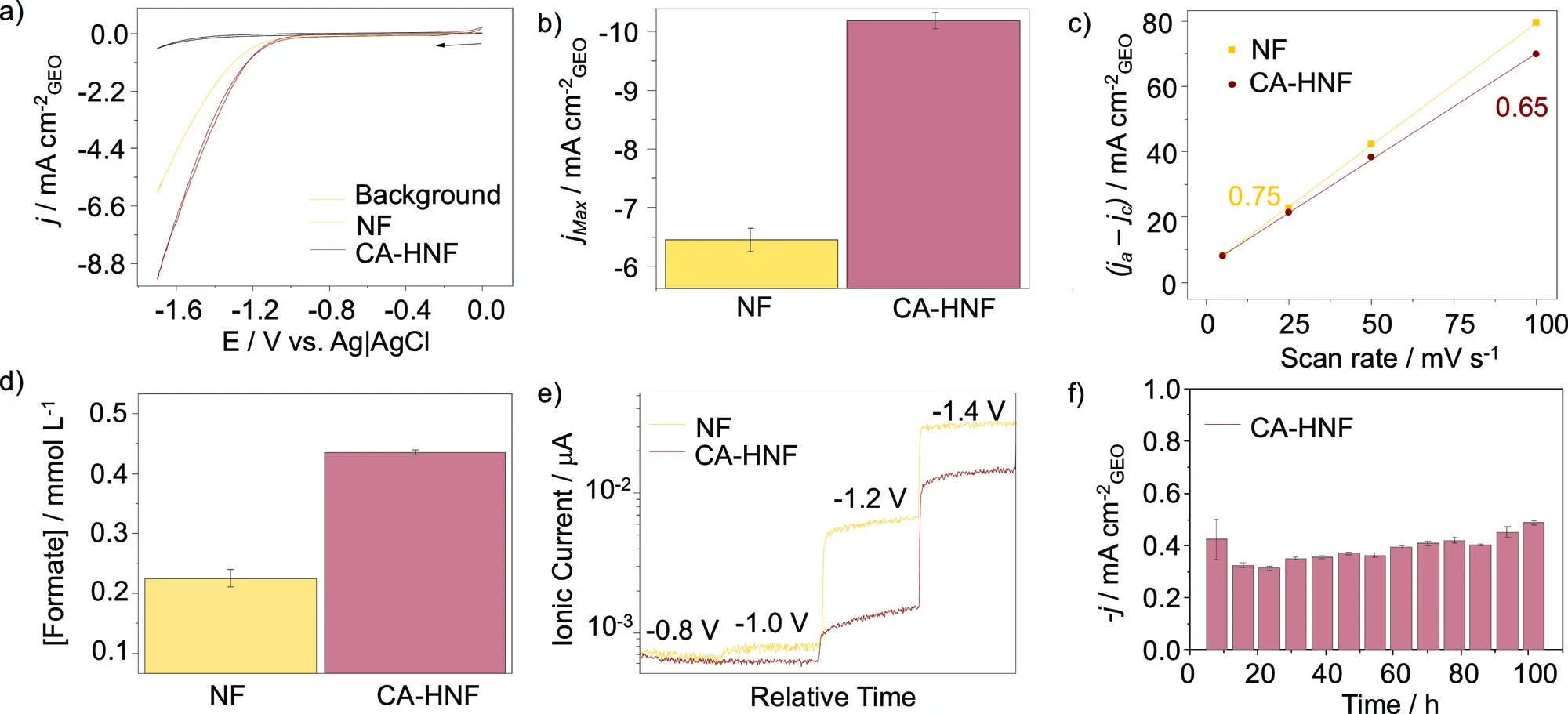
Bioelectrochemical characterization of NF and CA-HNF electrodes and analysis of CO_2_-reduction products. (a) Cyclic voltammograms recorded in 0.1 mol L^–1^ Tris-HCl buffer (pH 8) saturated with CO_2_, at a scan rate of 10 mV s^–1^, showing progressively enhanced biocatalytic current densities for the CA-HNF-based electrode; (b) maximum geometric current densities (*j*_Max_) extracted from the voltammograms, highlighting the increase of cathodic current with CA loading in the synthesis; (c) Double-layer capacitance plots obtained from Δ*j* as a function of scan rate, used to estimate electrochemically active surface area; slopes are indicated for each sample; (d) Formate concentration determined by HPLC analysis (at 210 nm) of the electrolyte collected after 5 h of electrolysis at −1.1 V vs Ag/AgCl.; (e) real-time DEMS profiles of H_2_ (*m*/*z* = 2) obtained during chronoamperometric potential steps in 0.1 M Tris-HCl buffer (pH 8) saturated with CO_2_, confirming earlier onset and higher H_2_ evolution for NF compared with CA-HNF; (f) stability performance of CA-HNF for CO_2_ reduction operated at −1.1 V vs Ag/AgCl over 100 h.

Although the onset potential remains within a narrow window for all samples (between −1.01 and −0.92 V vs Ag/AgCl), the current densities increase systematically for CA-containing samples, indicating that the observed enhancement arises from improved interfacial substrate availability rather than changes in intrinsic catalytic activity. Considering the standard potential for the CO_2_ reduction to HCOO^–^ (−0.63 V vs Ag/AgCl at pH 8.0 ^38^), the observed onset potentials correspond to overpotentials of approximately 0.29–0.38 V. Results for intermediate enzyme loadings are provided in Figure S7. The fact that CA-HNF005 and CA-HNF01 exhibit the highest current densities suggests that, at lower enzyme loadings, the structural features of the nanoflowers (more open architectures and larger accessible surface areas, as indicated by FE-SEM, and capacitance analysis in Figure S7c) may facilitate mass transport while still enabling the biochemical regeneration of CO_2_. This structural contribution, however, operates only among CA-containing samples: comparison with the enzyme-free NF electrode indicates that enzymatic activity, rather than surface area, remains the primary determinant of performance, since NF exhibits the largest Cdl of the entire series yet the lowest current density (−6.19 ± 0.16 mA cm^–2^) among all samples tested.

To further confirm that the enhanced electrochemical performance of CA-HNF arises specifically from catalytic CA activity, three independent control electrodes were prepared: a noncatalytic protein analog (BSA-HNF), heat-denatured CA-HNF (CA-HNF*), and CA-HNF in the presence of the CA inhibitor thiocyanate (SCN^–^). Chronoamperometric measurements (Figure S8) show that all three catalytically inactive controls converge to a common current density: BSA-HNF: −1.51 ± 0.01 × 10^–4^ A cm^–2^; CA-HNF*: −1.56 ± 0.01 × 10^–4^ A cm^–2^; CA-HNF + SCN^–^: −1.56 ± 0.004 × 10^–4^ A cm^–2^, well below that of active CA-HNF (−2.03 ± 0.004 × 10^–4^ A cm^–2^), regardless of the specific approach used to eliminate enzymatic function. Notably, the current densities of these controls were slightly lower than that of the NF electrode, which is consistent with the lower double-layer capacitance measured after protein incorporation during HNF synthesis, indicating a reduced relative electrochemical accessibility. In the absence of active CA, this structural effect is no longer offset by the enzyme-catalyzed increase in local CO_2_ concentration, resulting in lower catalytic currents.

Product analysis provided further insight into the role of CA in the biomimetic catalyst. Electrolyte samples collected after 5 h of electrolysis at a potential 100 mV more negative than the catalytic onset (-1.1 V vs Ag/AgCl) were analyzed by HPLC to quantify the concentrations of formate and acetate (Figures 5d and S7d). As summarized in Table S2, the total CO_2_RR faradaic efficiency increased from 16.15% for the NF electrode to 21.95%, 60.39%, and 69.70% for CA-HNF05, CA-HNF01, and CA-HNF005, respectively. This improvement was accompanied by an increase in the Faradaic efficiency toward both products. The Faradaic efficiency for formate increased from 8.46% for NF to 9.73%, 24.16%, and 28.94% for CA-HNF05, CA-HNF01, and CA-HNF005, respectively, while the Faradaic efficiency for acetate increased from 7.69% to 12.21%, 36.24%, and 40.75%. Consistent with these results, the average concentrations of formate increased from 0.24 to 0.35, 0.37, and 0.45 mmol L^–1^, whereas the concentrations of acetate increased from 0.22 to 0.44, 0.56, and 0.63 mmol L^–1^. The Faradaic efficiencies of the quantified products do not sum to 100%; therefore, the remaining fraction is reported as unassigned charge. Although hydrogen evolution is observed under these conditions, the unassigned charge cannot be attributed entirely to H_2_ without direct quantitative analytical evidence.

Importantly, the relative enhancement observed by hyperspectral micro-FTIR reflects a localized interfacial phenomenon, whereas current density and Faradaic efficiency are electrode-averaged quantities governed by multiple kinetic and transport processes. Consequently, local CO_2_ enrichment is not expected to translate linearly into an equivalent increase in total current or product selectivity.

These values are consistent with the enhanced cathodic currents observed in the voltammograms and DEMS analysis. Importantly, these results indicate that the enhancement in CO2RR product selectivity arises from improved interfacial CO_2_ availability rather than changes in the intrinsic catalytic properties of the material. In this context, the CA-HNF architecture demonstrates how microenvironment engineering can be integrated with electrocatalytic systems to modulate selectivity and efficiency.

DEMS measurements (Figure 5e) further clarified the effect of CA on gas-phase products. In electrodes containing CA, hydrogen evolution was only detected at potentials more negative than −1.0 V, whereas in the absence of the enzyme (NF), H_2_ formation began at significantly less negative potentials, around −0.8 V. This shift indicates that carbonic anhydrase modulates the potential range at which HER becomes competitive, delaying the onset of H_2_ evolution while CO_2_ reduction is occurring. Notably, no ionic current signal for the *m*/*z* = 15 (methane) or for *m*/*z* = 28 (carbon monoxide) fragments was observed throughout the experiments, indicating that CH_4_ or CO was not produced as a gas-phase product under the tested conditions. The FE analysis, in combination with DEMS measurements, indicates that CA contributes to modulating the competition between CO_2_ reduction and hydrogen evolution under the applied potentials.

The long-term performance of CA-HNF was additionally evaluated by chronoamperometry at −1.1 V vs Ag/AgCl for 100 h (Figure 5f). During the 100 h electrolysis, the current density gradually increased in magnitude from −0.3 to −0.4 mA cm^–2^. A possible explanation for this behavior is the continuous evolution of gas bubbles at the working electrode, which may progressively promote partial detachment of the hydrogel from the electrode surface, thereby exposing a larger fraction of the underlying bare graphite and leading to an increase in the measured current density.

To place these results in context, Table S2 compares the catalytic metrics of CA-HNFs with representative bioinspired and enzyme-based systems reported in the literature for CO_2_ reduction. For example, carbon felt electrodes modified with polypyrrole, Neutral Red, and dehydrogenases enable the formation of methanol with current densities of −1.5 to −3.0 mA cm^–2^ at −1.2 V vs Ag/AgCl and a FE of 10%.^39^ In contrast, a system based on formate dehydrogenase applied to formic acid production reached current densities of −6.97 mA cm^–2^ at −1.0 V vs Ag/AgCl in the production of 8.99 mM of HCOOH, but with only a FE of 12.74%.

These observations place CA-HNFs among competitive bioinspired approaches reported in the literature, demonstrating that enzymatic modulation of interfacial CO_2_ availability can achieve catalytic performances comparable to established enzyme-based pathways under near-neutral conditions. To place these findings in the broader context of contemporary carbon management, it is worth comparing this biohybrid approach with modern Gas Diffusion Electrodes (GDEs). While GDEs are highly effective at overcoming bulk solubility limits via gas-phase substrate delivery, they frequently suffer from localized carbonate precipitation and mass-transport failure when operated in near-neutral and alkaline media.^40^ The local pH increase driven by high consumption rates shifts the equilibrium, converting incoming molecular CO_2_ into inactive (bi)­carbonate species. Rather than simply presenting an alternative to displace conventional gas-phase cells, the CA-HNF architecture highlights the potential for designing *symbiotic technologies*. By integrating this biohybrid interface into the catalytic layers of existing GDE systems, the thermodynamic bottleneck of local carbonate accumulation can be turned into a distinct operational advantage. In this symbiotic configuration, instead of suffering from performance degradation due to localized alkalinity, the system would actively utilize the generated bicarbonate pool as a continuous, *in situ* substrate reservoir. The rapid enzymatic relaxation of the electrolyte within the nanoconfined GDE pores would sustain a high localized CO_2_ flux under mild conditions, bridging the gap between biological carbon fixation and high-power electrochemical engineering.

## 4 Conclusion

In summary, we have established a bioinspired reaction–diffusion nanoreactor that overcomes the fundamental mass-transport limitations of aqueous CO_2_ reduction. By integrating carbonic anhydrase within hierarchical copper phosphate nanoflowers, we successfully mimicked the CO_2_-concentrating mechanism of natural carboxysomes, effectively transforming the catalytic interface into a self-sustaining CO_2_ reservoir.

The combination of state-of-the-art hyperspectral micro-FTIR imaging and numerical modeling provided spatiotemporal evidence that the in situ enzymatic dehydration of bicarbonate elevates local CO_2_ activity. This localized enrichment effectively collapses the characteristic diffusion boundary layer from micrometric to nanometric dimensions, ensuring a saturated substrate environment even under transport-limited conditions.

Our findings demonstrate that managing the interfacial chemical microenvironment is as critical as engineering the intrinsic kinetics of the active sites. This study provides a compelling framework for the design of hybrid electrocatalytic systems, where the synergistic coupling of biocatalysis and structural confinement enables high-performance electrosynthesis in near-neutral media. Ultimately, this work offers a strategy for developing sustainable, biohybrid technologies, bridging the gap between biological efficiency and industrial scalability in carbon utilization.

## Supplementary Material



## Notes

Experimental details regarding chemicals and reagents; protocol for the determination of carbonic anhydrase loading via UV–Vis spectroscopy; detailed bioelectrode assembly and electrochemical cell configurations; detailed high-resolution XPS analysis; methodology and mathematical modeling parameters for custom Python scripts simulating micro-FTIR accumulation grids; electrochemical performance analysis across varying enzyme loadings (cyclic voltammograms, capacitive current plots, and HPLC chromatograms); relative electrochemically active surface area (ECSA) estimation via double-layer capacitance protocol; and chromatographic calibration curves for formate and acetate quantification alongside the Faradaic efficiency equations (PDF)

## †

Institute of Chemistry, São Paulo State University (UNESP), 14800-900 Araraquara, SP, Brazil
