# Supplementary material for: Decoupling CO2 Availability from Bulk Solubility via Biohybrid Reaction–Diffusion Nanoconfinement
Source: J Am Chem Soc. 2026 Aug 31;148(36):39054–66. doi: 10.1021/jacs.6c12039 (PMC13587671; doi:10.1021/jacs.6c12039)
Supplement: Supplementary file 1 [file ja6c12039_si_001.pdf]

Supporting Information

Decoupling CO_2 Availability from Bulk Solubility via Biohybrid Reaction-Diffusion Nanoconfinement

Fhysmélia F. Albuquerque, Artur C. Souza, Matheus S. Corsino, Lucas D. Paquini, Graziela C. Sedenho, Rafael N. P. Colombo, Fabio H. B. Lima, Frank N. Crespilho

1. Materials. 1
2. Bioelectrode preparation and cell configuration 2
3. Determination of CA loading in nanoflowers 2
4. XPS Analysis 3
5. Carbonic anhydrase activity assay (Wilbur–Anderson method) 4
6. Simulation of micro-FTIR CO_2 accumulation at CA-HNF interfaces 5
7. Electrochemical performance of CA-HNF materials with varying enzyme loadings 8
8. Formate and acetate quantification and Faradaic efficiency 9
9. Control electrodes for mechanistic validation 10
10. Electrochemical surface area (ECSA) estimation of NF and CA-HNFs 10
11. Hydrogel thickness, surface morphology, and ionic conductivity 11
12. Supplementary Figures 12
13. Supplementary Tables 19

1. Materials.

All reagents were of analytical grade and used without further purification. Carbonic anhydrase (from bovine erythrocytes, in the lyophilized powder form), glutaraldehyde (≥25%), copper sulfate (98%), sodium hydroxide (98%), sulfuric acid (98%), and Tris(hydroxymethyl)aminomethane were acquired from Sigma-Aldrich. Agarose was obtained from Kasvi, and Sodium sulfate (99%), hydrochloric acid (37%), sodium

phosphate dibasic anhydrous (98%), sodium phosphate monobasic monohydrate (98%), and sodium chloride (99%) were supplied by Êxodo Científica. HEDGE graphite electrodes were obtained from Alpha Chemicals.

2. Bioelectrode preparation and cell configuration

For bioelectrode fabrication, the biomimetic catalysts were dispersed in a precursor solution of agarose hydrogel (1.5 mg mL^{-1}) containing glutaraldehyde (1.25 %). The suspension was drop-cast onto pretreated HEDGE electrodes and dried under vacuum for 60 min to ensure proper adhesion of the composite to the conductive substrate (**Figure S1b**). This procedure was adapted from previous work[2]. For electrochemical measurements, the working electrode was the HEDGE electrode modified with an agarose hydrogel containing the biomimetic catalyst; Ag/AgCl (saturated) served as the reference electrode, and a platinum plate was used as the counter electrode (**Figure S1c**). All electrodes were obtained from the same commercial source, cut to identical dimensions, and subjected to the same pretreatment procedure. Catalyst mass, hydrogel composition, deposited volume, geometric area, and drying conditions were maintained constant. Electrochemical measurements were performed using independently prepared electrodes in triplicate.

3. Determination of CA loading in nanoflowers

The enzyme content remaining in the supernatant after synthesis was quantified by UV-Vis spectroscopy, measuring the absorbance at 280 nm and using the supernatant of the control synthesis (0 mg mL^{-1} of CA) as the blank. The concentration of CA in the supernatant was calculated using the $E_{1\%,280} = 19.0$ according to[1]:

$$[CA]_{supernatant} = \frac{Abs_{280}}{E_{1\%}} \times 10\ (mg\ mL^{-1}) \quad \textbf{(Eq S1)}$$

The enzyme loading was evaluated by quantifying the residual CA in the supernatant after synthesis. As shown in **Table S1**, a clear dependence on the initial enzyme concentration was observed. For CA-HNF05, a relatively high absorbance (0.7960 ± 0.0159) and residual concentration (0.4190 mg mL^{-1}) were measured in the supernatant, corresponding to a loading of 16.2%. In contrast, decreasing the initial CA concentration led to a substantial increase in incorporation. CA-HNF01 exhibited a loading of 24.3%, while CA-HNF005 showed a markedly higher loading of 92.8%, with minimal residual enzyme detected in solution. These results indicate that lower initial enzyme concentrations favor more efficient incorporation into the nanoflower structure, likely due to reduced competition for coordination sites during nucleation and growth.

4. XPS Analysis

The O 1s spectrum (**Figure S3a**) was deconvoluted into three main components centered at 530.5, 531.5, and 532.0 eV. The lowest binding energy contribution at 530.5 eV is assigned to Cu-O-P environments[3], characteristic of copper phosphate species. The component at 531.5 eV is attributed to C=O and P=O groups[4]. The higher binding energy peak at 532.0 eV is assigned to carboxyl (-COOH) groups[5]. The C 1s spectrum (**Figure S3b**) was fitted with three main components located at 283.8, 284.9, and 286.4 eV. The peak at 283.8 eV is attributed to C-C bonds. The contribution at 284.9 eV is assigned to C-N bonds. The higher binding energy component at 286.4 eV corresponds to oxidized carbon species, including C=O, C-OH, C-O-C, and O=C-NH groups[5]. It should be noted that the functional groups identified in the high-resolution O 1s and C 1s spectra are not exclusively associated with the immobilized enzyme. These contributions may also originate from oxygen- and carbon-containing functionalities present on the HEDGE substrate. Therefore, the observed O 1s and C 1s components reflect the combined

contributions of the copper phosphate phase, the carbonic anhydrase, and the underlying graphite surface. Additionally, the high-resolution P 2p spectrum (**Figure S3c**) is dominated by contributions assigned to phosphate species, with components at 133.1 eV and 132.2 eV, together with an additional peak at 131.4 eV, attributed to P-O-C bonding[6]. The Cu 2p spectrum of the sample after 2 h of electrolysis at -1.1 V vs Ag/AgCl (**Figure S3d**) was analyzed to evaluate changes in the copper chemical state under the applied conditions. The Cu $2p_{3/2}$ region was deconvoluted into contributions centered at ~932.2–932.6 eV and ~933.4–935.1 eV, assigned to Cu^0/Cu^+ and Cu^{2+} species, respectively. The relative fractions were obtained from the areas of the fitted components. The as-prepared sample shows 7.74% Cu^0/Cu^+ and 92.25% Cu^{2+}, whereas after 2 h of electrolysis, these values change to 34.89% and 65.11%, respectively, indicating the formation of reduced copper species at the surface under the applied conditions.

5. Carbonic anhydrase activity assay (Wilbur–Anderson method)

CA activity was determined using the classical electrometric method of Wilbur and Anderson. Briefly, CO_2-saturated water was prepared by bubbling CO_2 gas through ice-cold deionized water for 30 min prior to the assay. A 0.012 mol L^{-1} Tris·HCl buffer (pH 8.3) was placed in a small vessel immersed in an ice bath (0 °C) under gentle stirring, and the initial pH was confirmed using a calibrated pH electrode. For the uncatalyzed control, a fixed volume of CO_2-saturated water was added to the buffer, and the pH decay was monitored over time. For catalyzed reactions, free CA or CA-HNF005 (using equal total masses of material) was added to the Tris buffer prior to the addition of CO_2-saturated water. In all cases, pH was recorded at 0, 10, 20, 40, 60, and 80 s after mixing, and the time required for the pH to drop from 8.3 to 6.3 was determined by linear interpolation between the two closest measured time points. All measurements were performed in

triplicate (n = 3) at 0 °C. The uncatalyzed reaction required $T_0 = 58.1 \pm 2.5$ s to reach pH 6.3. In the presence of free CA, this time was reduced to $T = 21.0 \pm 1.6$ s, while CA-HNF005 reduced it to $T = 26.1 \pm 6.8$ s **(Figure S4).** Expressed as Wilbur–Anderson units [$WAU = (T_0 - T)/T$], this corresponds to 1.77 for free CA and 1.23 for CA-HNF005, indicating that the immobilized enzyme retains approximately 69% of the catalytic enhancement observed for the free enzyme. The larger relative variability observed for CA-HNF005 (coefficient of variation ≈ 26%, versus ≈ 8% for free CA) likely reflects the heterogeneous nature of the solid suspension, in contrast to the homogeneous free-enzyme solution.

6. Simulation of micro-FTIR CO_2 accumulation at CA-HNF interfaces

Micro-FTIR simulations were designed to reproduce the spatial resolution, focal volume, and field of view used in the experimental measurements. The simulated domain consisted of a two-dimensional Cartesian grid representing a micro-FTIR mapping area, with spatial step sizes in the micrometre range, consistent with typical FPA-based acquisitions. The total simulated field extended over several hundred micrometres to include both the CA-HNF particle and the surrounding area.

CA-HNF was modeled as a single irregular particle with an effective diameter of approximately 100 μm, in agreement with the experimentally observed agglomerates. To mimic realistic morphology, the particle boundary was defined by a nominal circular geometry with superimposed low-amplitude spatial perturbations, reproducing edge roughness and local anisotropy commonly observed in optical micrographs. The particle was embedded in a continuous D_2O-based phase representing the bicarbonate solution.

The local concentration of molecular CO_2 (denoted CO_2^*) was determined from the thermodynamic equilibrium between bicarbonate and CO_2. For a bulk bicarbonate

concentration of 0.1 M, the equilibrium CO_2* concentration was estimated using the Henderson–Hasselbalch relationship, assuming a pKa ≈ 6.35. Depending on pH, this yields equilibrium CO_2* concentrations in the 10^{-4}-10^{-3} M range. These values were used as the bulk reference concentration far from the CA-HNF particle.

Within the CA-HNF region and its immediate vicinity, the CO_2* concentration was locally enhanced to account for the combined effects of CA activity, reduced effective diffusion, and interfacial confinement. Importantly, the simulations do not assume any shift in thermodynamic equilibrium induced by CA; rather, CA is treated as a kinetic accelerator that rapidly maintains local equilibrium under conditions of spatial heterogeneity. A smooth spatial gradient was imposed at the particle-solution boundary to represent diffusion-controlled relaxation toward bulk concentrations.

The role of CA was introduced implicitly through the assumption of rapid equilibration. Given the exceptionally high turnover frequency of CA, the enzymatic interconversion between CO_2 and bicarbonate is effectively instantaneous on the timescale of micro-FTIR acquisition. As a result, the local CO_2 concentration field is governed by equilibrium thermodynamics and mass transport rather than reaction kinetics. This justifies the use of steady-state concentration profiles in the simulations.

Local CO_2* concentrations were converted into simulated micro-FTIR absorbance at 2350 cm^{-1} using the Beer–Lambert relationship:

$$A_{2350} = \varepsilon \cdot l \cdot [CO_2^*] \qquad \textbf{(Eq S2)}$$

Where ε is the effective absorption coefficient of the CO_2 ν_3 mode and l is the optical path length within the micro-FTIR focal volume. Because absolute values of ε and l are generally unknown in heterogeneous micro-FTIR measurements, all simulated

absorbance maps were normalized by the maximum equilibrium absorbance, yielding dimensionless quantities $A_{2350}/A_{2350,eq}$. This normalization procedure mirrors the experimental analysis and allows direct comparison between simulation and experiment without adjustable scaling factors.

Two-dimensional absorbance maps were generated by assigning a normalized absorbance value to each spatial pixel in the simulation grid. The maps were rendered using the same color palette as the experimental data, with low CO_2 concentrations represented in blue and progressively higher concentrations mapped through green, yellow, and orange to pink/magenta at the maximum. This ensured direct visual comparability between simulated and experimental micro-FTIR images. Three-dimensional surface representations were constructed from the same normalized absorbance data, with the z-axis corresponding to $A_{2350}/A_{2350,eq}$. These plots emphasize the interfacial enrichment of CO_2 at CA-HNF and the rapid decay toward bulk solution values.

All simulation parameters were chosen to reflect experimentally accessible quantities, including particle size, spatial resolution, and bicarbonate concentration. No fitting to experimental absorbance intensities was performed beyond normalization to equilibrium values. The close agreement between simulated and experimental micro-FTIR maps, both in spatial localization and relative intensity distribution, supports the interpretation that the experimentally observed $\nu_3(CO_2)$ signal originates from genuine interfacial accumulation of molecular CO_2 at CA-HNF, sustained by carbonic anhydrase activity.

All simulations and data visualizations were performed using custom scripts written in Python (version 3.x), employing standard scientific libraries for numerical computation and plotting. Spatial grids and concentration fields were generated using

NumPy, while two- and three-dimensional visualizations were produced with Matplotlib, including its mplot3d toolkit for surface rendering. Custom colormaps were defined to exactly reproduce the experimental micro-FTIR color palette, ensuring direct visual comparability between simulated and measured maps. The CA-HNF particle geometry was modeled explicitly within the spatial grid, incorporating controlled boundary perturbations to mimic experimentally observed morphological irregularities. Equilibrium CO_2 concentration fields were calculated analytically from thermodynamic relations and imposed as steady-state solutions, with spatial gradients introduced via exponential decay functions representing diffusion-limited relaxation into the bulk solution. Conversion from concentration to simulated IR absorbance was performed using Beer-Lambert scaling, followed by normalization to the equilibrium maximum absorbance ($A_{2350}/A_{2350,eq}$), thereby eliminating dependence on unknown optical path length or absolute absorption coefficients. All figures were exported in high-resolution raster formats (600 dpi PNG) and scalable vector graphics (SVG).

7. Electrochemical performance of CA-HNF materials with varying enzyme loadings

The electrochemical behavior of CA-HNF materials prepared with different enzyme loadings (CA-HNF005, CA-HNF01, and CA-HNF05) is shown in **Figure S7**. The cyclic voltammograms (**Figure S7a**) indicate that the maximum current density (J_{max}) at -1.7 V increases as the enzyme concentration used during synthesis decreases (**Figure S7b**). CA-HNF005 and CA-HNF01 exhibited the highest current densities (-9.34 ± 0.12 and -8.33 ± 0.20 mA cm^{-2}, respectively), while CA-HNF05 showed a lower value (-7.23 ± 0.04 mA cm^{-2}), compared to -6.19 ± 0.16 mA cm^{-2} for the enzyme-free NF. The double-layer capacitance (C_{dl}), estimated from the slope of the capacitive current as a function of scan

rate (**Figure S7c**), also follows a systematic trend, with the highest slope observed for NF, followed by CA-HNF005, CA-HNF01, and CA-HNF05. The methodology used to determine C_{dl} is described in Section 10 of the Supporting Information. HPLC analysis (**Figure S7d and Table S2**) revealed a similar trend in product formation, with formate production increasing from 0.24 ± 0.02 mmol L^{-1} for NF to 0.35 ± 0.04, 0.37 ± 0.05, and 0.45 ± 0.005 mmol L^{-1} for CA-HNF0.5, CA-HNF0.1, and CA-HNF0.05, respectively. Acetate production followed a similar trend, increasing from 0.22 ± 0.21 mmol L^{-1} for NF to 0.44 ± 0.03, 0.56 ± 0.05, and 0.63 ± 0.04 mmol L^{-1} for CA-HNF0.5, CA-HNF0.1, and CA-HNF0.05, respectively. These results confirm that enzyme incorporation enhances catalytic performance, with the best results observed at lower enzyme loadings, likely due to improved structural features that favor mass transport while maintaining enzymatic activity.

8. Formate and acetate quantification and Faradaic efficiency

The calibration curves shown in **Figure S7e and S7f** were used to convert chromatographic peak areas into formate and acetate concentrations, respectively. Standards of known concentration were analyzed under the same chromatographic conditions, and the resulting linear regression equation was applied to calculate the concentrations reported in **Table S2**. The Faradaic efficiency (FE) was calculated to quantify the fraction of the total electrical charge applied that was converted into products during the CO_2 bioelectroreduction. The FE was calculated according to **Eq S4**:

$$FE_{liquid}(\%) = \frac{N \times n \times F}{Q_{total}} \times 100 \quad \textbf{(Eq S4)}$$

Where,

$$Q = \int_0^{18000} i(t)dt \quad \textbf{(Eq S5)}$$

N is the amount of product (in mol), n is the number of electrons involved in the electrochemical formation of one molecule of formate ($2e^-$) or acetate ($8e^-$) from CO_2, and F is the Faraday constant (96485 C mol^{-1}).

9. Control electrodes for mechanistic validation

To confirm that the enhanced electrochemical performance of CA-HNF arises specifically from catalytically active carbonic anhydrase, three control electrodes were prepared, each eliminating CA catalytic activity through a distinct mechanism, while preserving the same hydrogel composition, catalyst loading, geometric area, and immobilization procedure used for the active CA-HNF electrode **(Figure S8)**. First, a non-catalytic protein analog was prepared by substituting CA with bovine serum albumin (BSA) at the same concentration used for CA-HNF005 synthesis (0.05 mg mL^{-1}), following the same nanoflower synthesis protocol described (20 μL of 120 mmol L^{-1} $CuSO_4$ added to 3 mL PBS, 0.1 mol L^{-1}, pH 7.4, containing BSA). For heat-denatured CA-HNF (CA-HNF*), native CA-HNF005 was thermally denatured after synthesis by heating inside the hydrogel at 100 °C for 10 min, in order to eliminate enzymatic activity while preserving the nanoflower architecture already formed. Finally, CA-HNF005 electrodes were incubated with potassium thiocyanate (KSCN, 0.1 mol L^{-1}) for 15 min prior to electrochemical measurements, to chemically inhibit the CA active site via competitive binding to the catalytic zinc center. All electrodes (NF, CA-HNF, BSA-HNF, CA-HNF*, and CA-HNF + SCN^-) were evaluated by chronoamperometry at -1.1 V vs. Ag/AgCl, under CO_2-saturated electrolyte, using the same three-electrode configuration described (Ag/AgCl reference, platinum counter electrode).

10. Electrochemical surface area (ECSA) estimation of NF and CA-HNFs

The ECSA of the electrodes was evaluated by measuring the electrochemical double-layer capacitance (C_{dl})[7]. Cyclic voltammograms were recorded in a 0.1 mol L^{-1} Tris-HCl buffer supporting electrolyte at scan rates ranging from 5 to 100 mV s^{-1}, within a non-faradaic potential window between -0.46 and -0.44 V vs Ag/AgCl. The corresponding voltammograms are shown in **Figure S9**. The double-layer capacitance was obtained by extracting the anodic (j_a) and cathodic (j_c) capacitive current densities at a fixed potential within the selected window (-0.45 V vs Ag/AgCl). The difference between these current densities ($\Delta j = j_a - j_c$) was plotted as a function of the scan rate. The slope of the resulting linear fit, divided by two, was taken as the C_{dl} value for each electrode. The ECSA can be expressed according to **Eq S3**:

$$ECSA = \frac{C_{dl}}{C_s} \quad \textbf{(Eq S3)}$$

Where C_s is the specific capacitance of the electrode material. In the present study, an absolute value of Cs is not available for the hybrid nanoflower electrodes. However, since all samples share the same electroactive base material the specific capacitance can be assumed to be constant across the electrode series. As a result, the denominator in **Eq S3** is identical for all samples, and the ECSA scales proportionally with the measured C_{dl} values. Therefore, C_{dl} was used as a relative descriptor to compare the electrochemically accessible surface areas of the different electrodes.

11. Hydrogel thickness, surface morphology, and ionic conductivity

The agarose/glutaraldehyde hydrogel exhibited a dry-state thickness of L ≈ 10 µm, determined from cross-sectional measurements of the deposited film (**Figure S10).** Surface morphology was examined by AFM at multiple magnifications **(Figure S11),** since the hydrogel exhibited features spanning nanometric to micrometric length scales.

Apparent pore diameters were calculated as d = 2r from the radial distance between the center and boundary of each depression, using the AFM scale bar for pixel-to-distance calibration (n = 9 depressions, 6 fields of view; **Table S3**). Two populations were observed: submicrometer depressions (d = 0.271 ± 0.101 μm, n = 5) and larger micrometer-scale openings (d = 2.02 ± 1.16 μm, n = 4), indicating a heterogeneous, multiscale surface rather than a single pore population. These values represent apparent dry-state dimensions and may differ from the hydrated, operating-condition morphology due to agarose swelling.

Ionic conductivity was evaluated by electrochemical impedance spectroscopy, fitting spectra to an Rs–(CPE||Rct) equivalent circuit, where Rs represents the high-frequency series resistance associated with ionic migration through the film. Apparent conductivity was calculated as $\sigma_{app} = \frac{L}{Rs \times A}$, using the measured film thickness (L = 10 × 10^{-4} cm) and electrode geometric area (A = 0.556 cm^2). The average series resistance was Rs = 82.9 ± 0.2 Ω **(Table S4),** giving σ_{app} = (2.170 ± 0.006) × 10^{-5} S cm^{-1} (21.70 ± 0.06 μS cm^{-1}).

While this apparent conductivity is lower than that of the bulk electrolyte, and the measured pore dimensions reflect the dry state rather than the hydrated, operating condition, the same hydrogel formulation, thickness, and preparation protocol were used across all electrodes compared in this study. Any associated transport resistance is therefore common to NF, CA-HNF, and the catalytically inactive controls, and cannot account for the enhanced performance observed specifically for catalytically active CA-HNF.

12. Supplementary Figures

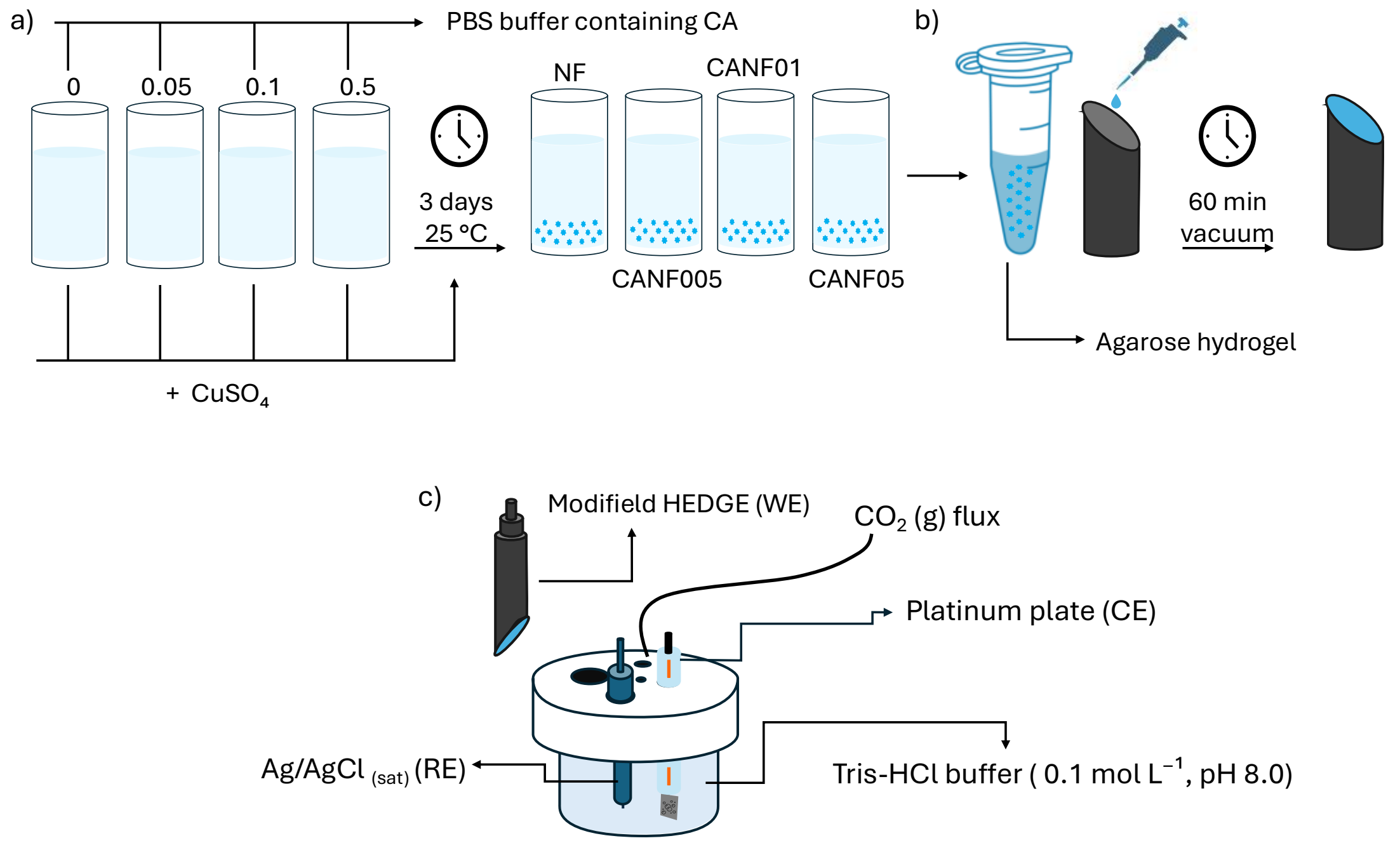

Figure S1. Scheme of (a) CA-HNFs synthesis; (b) bioelectrode preparation and (c) cell configuration

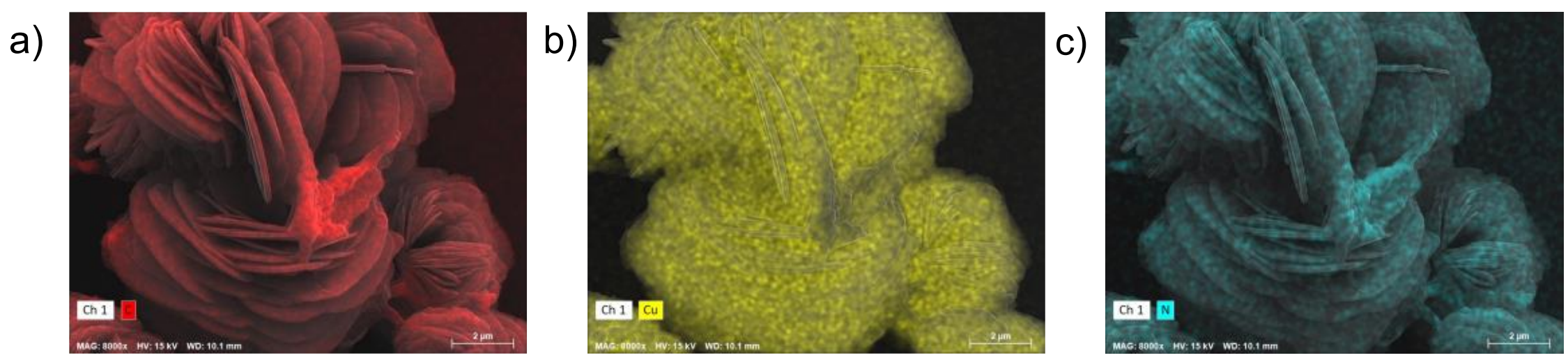

Figure S2. EDS of the individual signals of C (red), Cu (yellow) and N (cyan) within the hybrid structures.

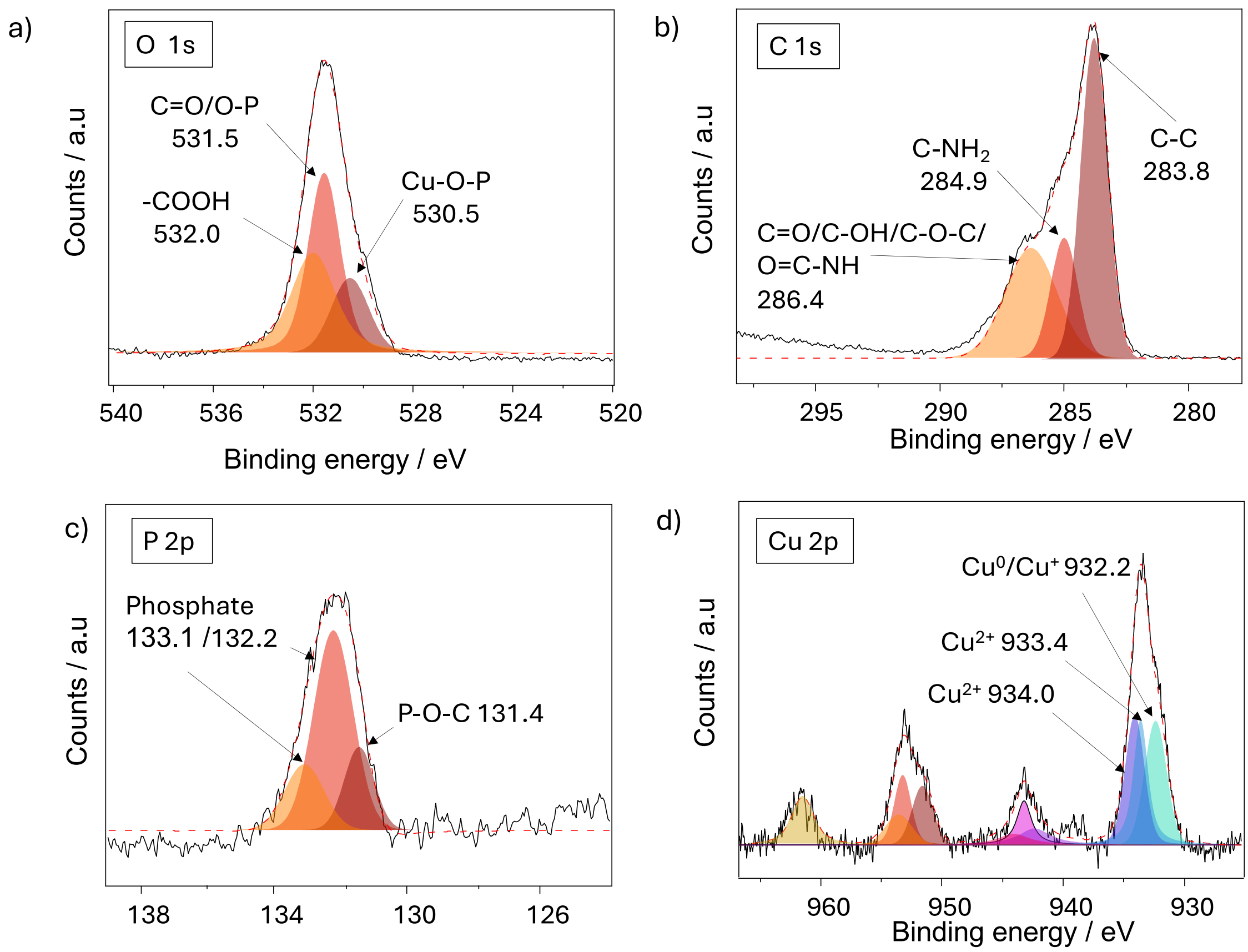

Figure S3. High-resolution XPS spectra in (a) O 1s, (b) C 1s and (c) P 2p regions; (d) Cu 2p after 2 h of electrolysis. Raw spectra are shown in solid lines and the cumulative fit peak in dashed lines.

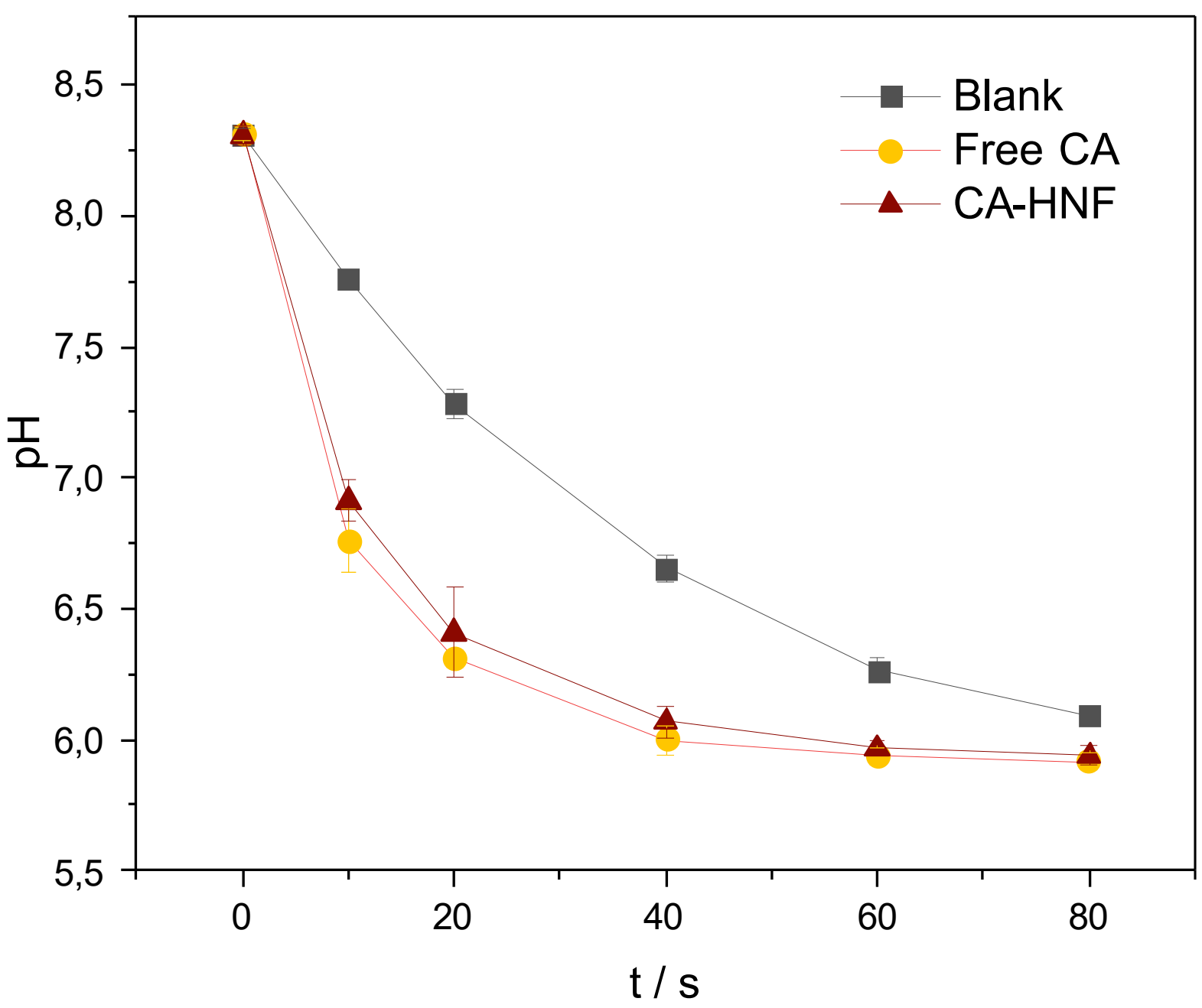

Figure S4. pH decay curves for the Wilbur-Anderson assay: blank, free CA, and CA-HNF, used to determine enzymatic activity retention after immobilization.

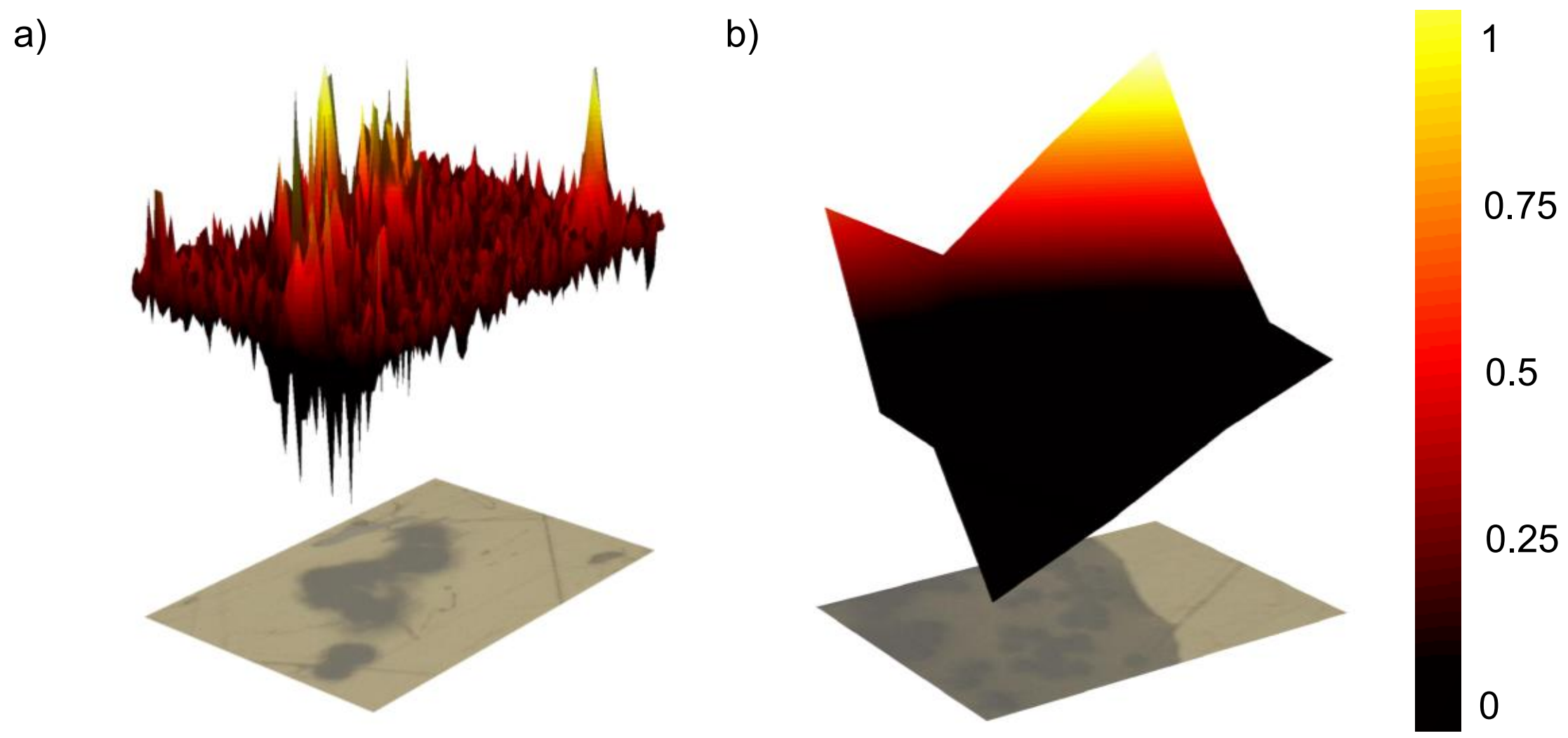

Figure S5. Micro-FTIR analysis of (a) CA-HNF and (b) NF in the presence of aqueous bicarbonate solution; 3D chemical maps of integrated CO_2 band intensity (2350 cm^{-1}) overlaid on the optical image and normalized color scale bar.

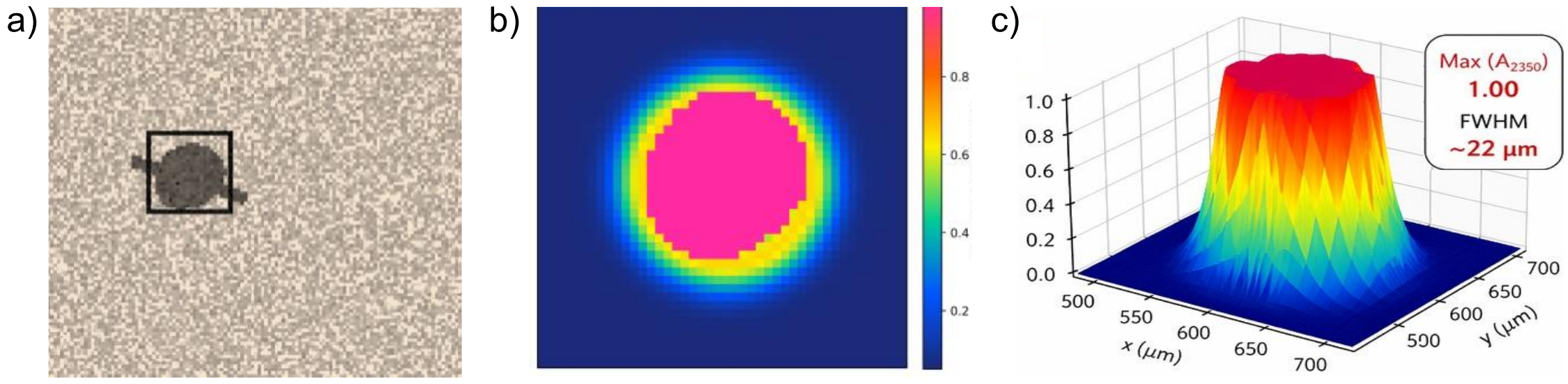

Figure S6. Simulated (a) optical CA-HNF image, (b) 2D and (c) 3D-FTIR maps showing a localized CO_2 accumulation for CA-HNF.

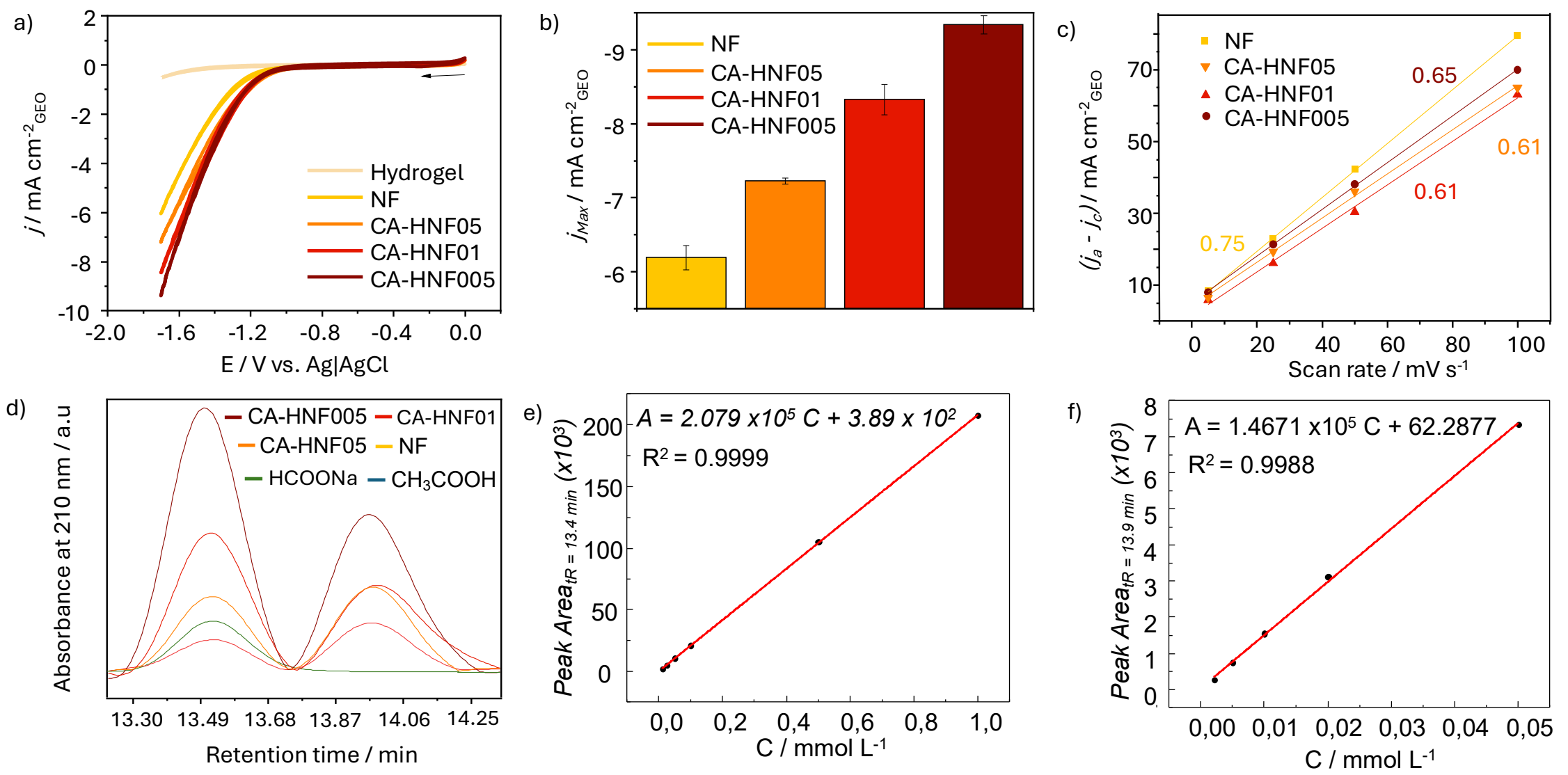

Figure S7. (a) Cyclic voltammograms recorded in 0.1 mol L-1 Tris-HCl buffer (pH 8) saturated with CO_2, at a scan rate of 10 mV s^{-1}, showing progressively enhanced biocatalytic current densities for the CA-HNF-based electrodes; (b) Maximum geometric current densities (jMax) extracted from the voltammograms, highlighting the increase of cathodic current with lower initial CA loading in the synthesis; (c) Double-layer capacitance plots obtained from Δj as a function of scan rate, used to estimate electrochemically active surface area; slopes are indicated for each sample; (d) HPLC chromatograms of the electrolyte collected after 5 h of electrolysis at -1.1 V vs. Ag/AgCl, monitoring formate and acetate at 210 nm; HPLC calibration curve for (e) formate and (f) acetate quantification;

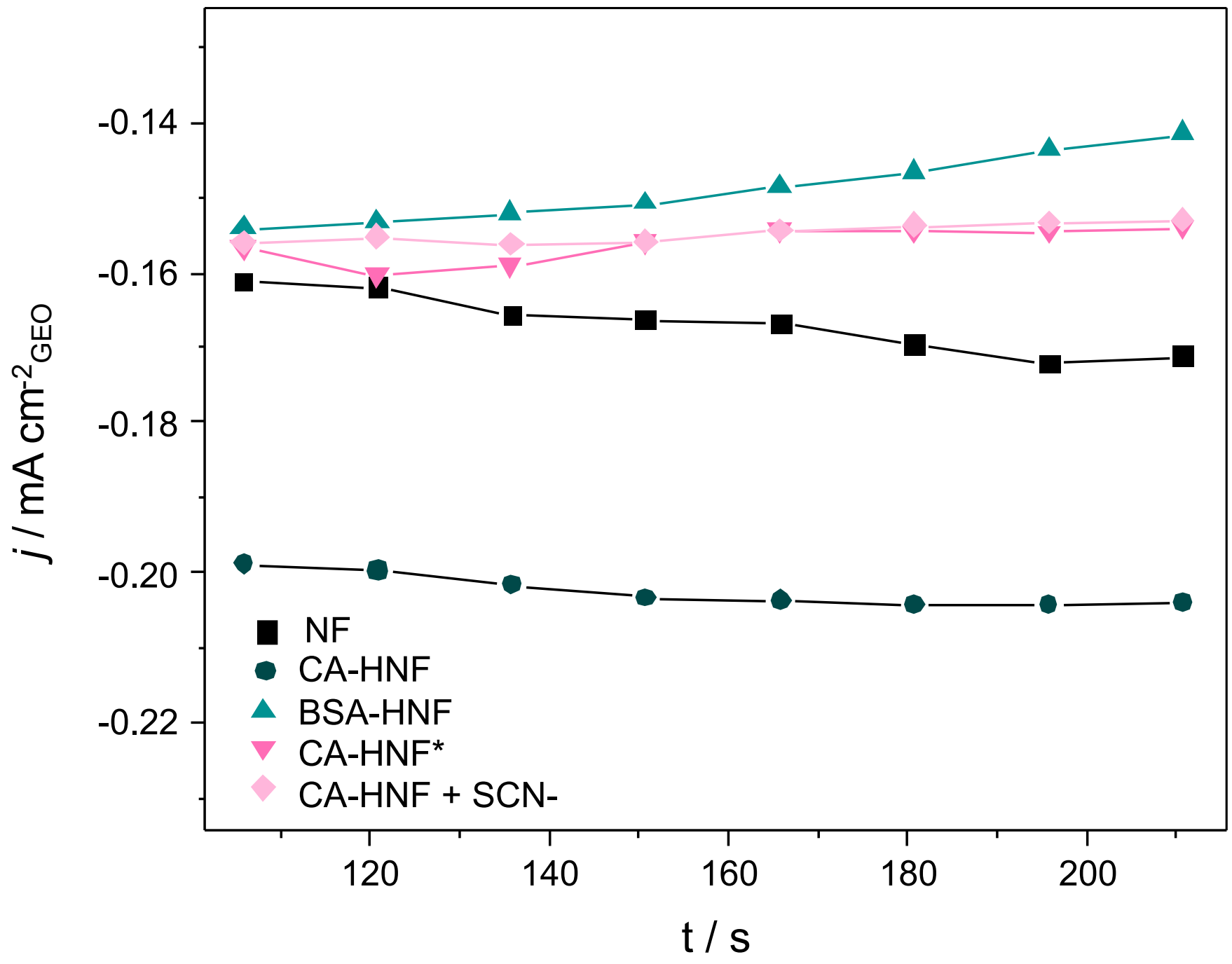

Figure S8. Electrolysis performed at -1.1 V vs Ag/AgCl for NF (black), CA-HNF (dark green), BSA-HNF (light green), heat-denatured CA-HNF (pink), and CA-HNF in the presence of SCN^- (light pink).

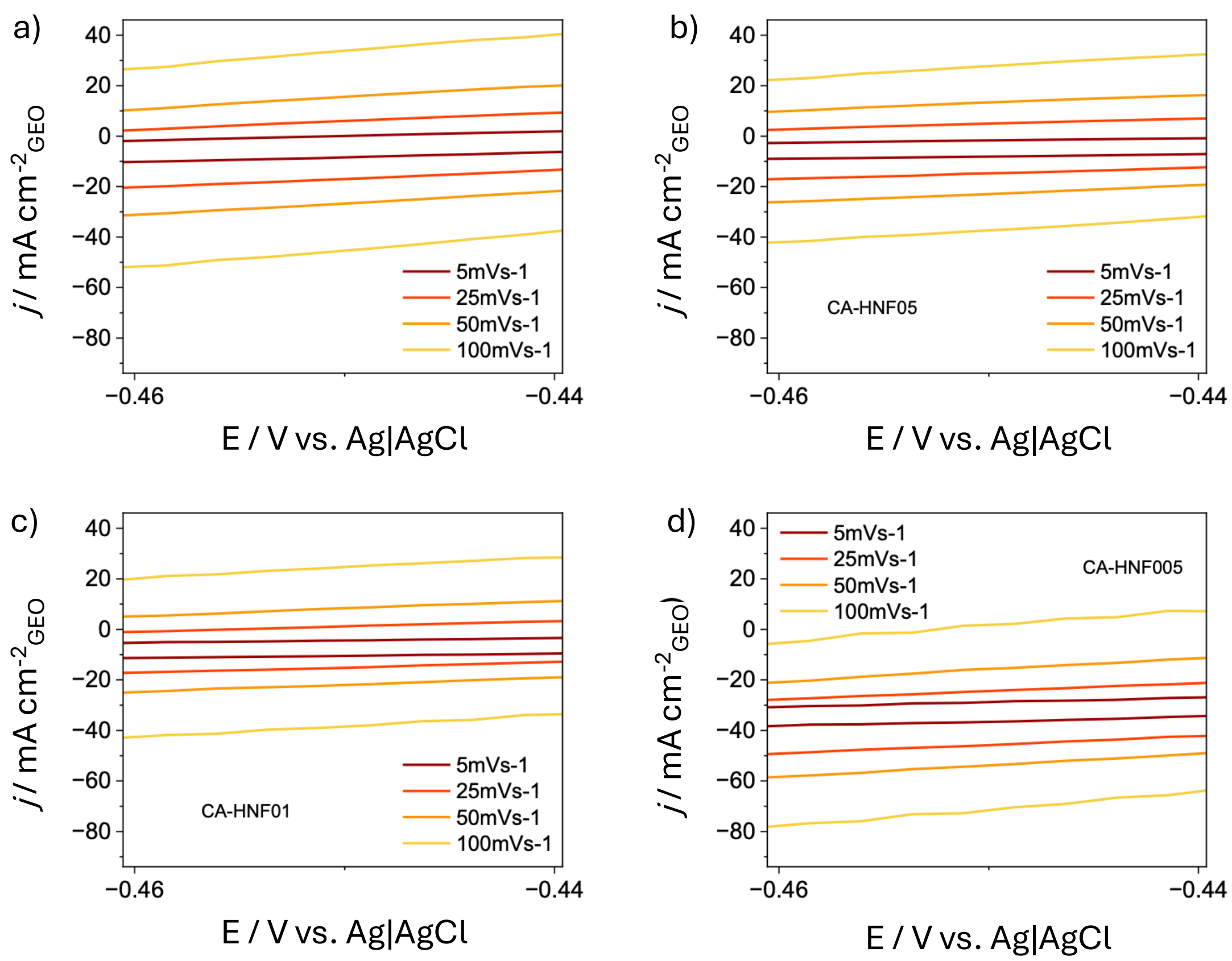

Figure S9. CVs of (a) NF, (b) CA-HNF05, (c) CA-HNF01 and (d) CA-HNF005 used for the determination of the double-layer capacitance.

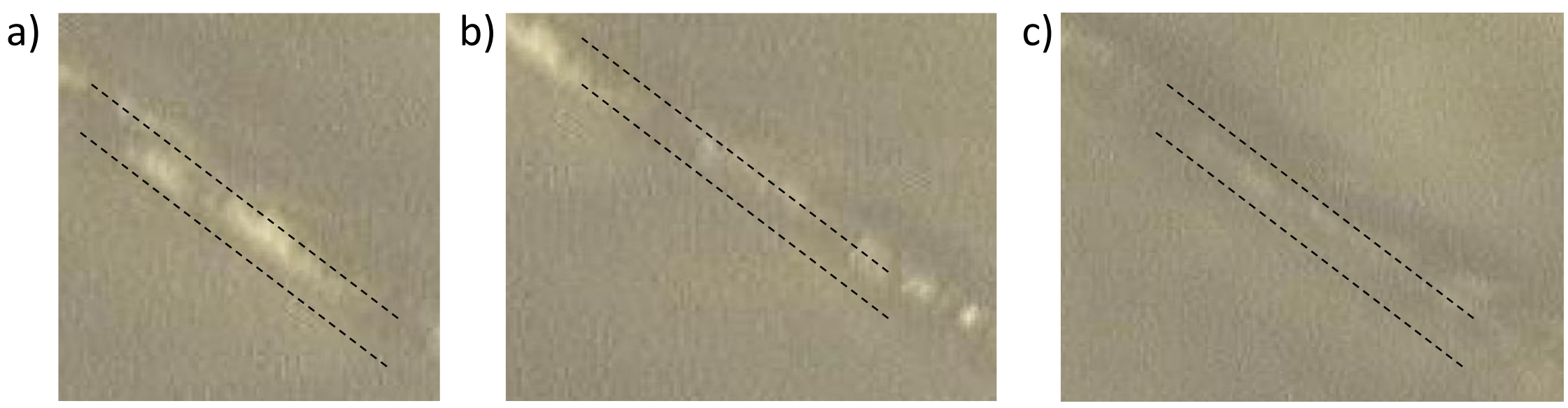

Figure S10. Optical microscopy images showing the hydrogel layer used for thickness measurements. The dashed lines delimit a distance of 10 μm, corresponding to the thickness measured for the hydrogel.

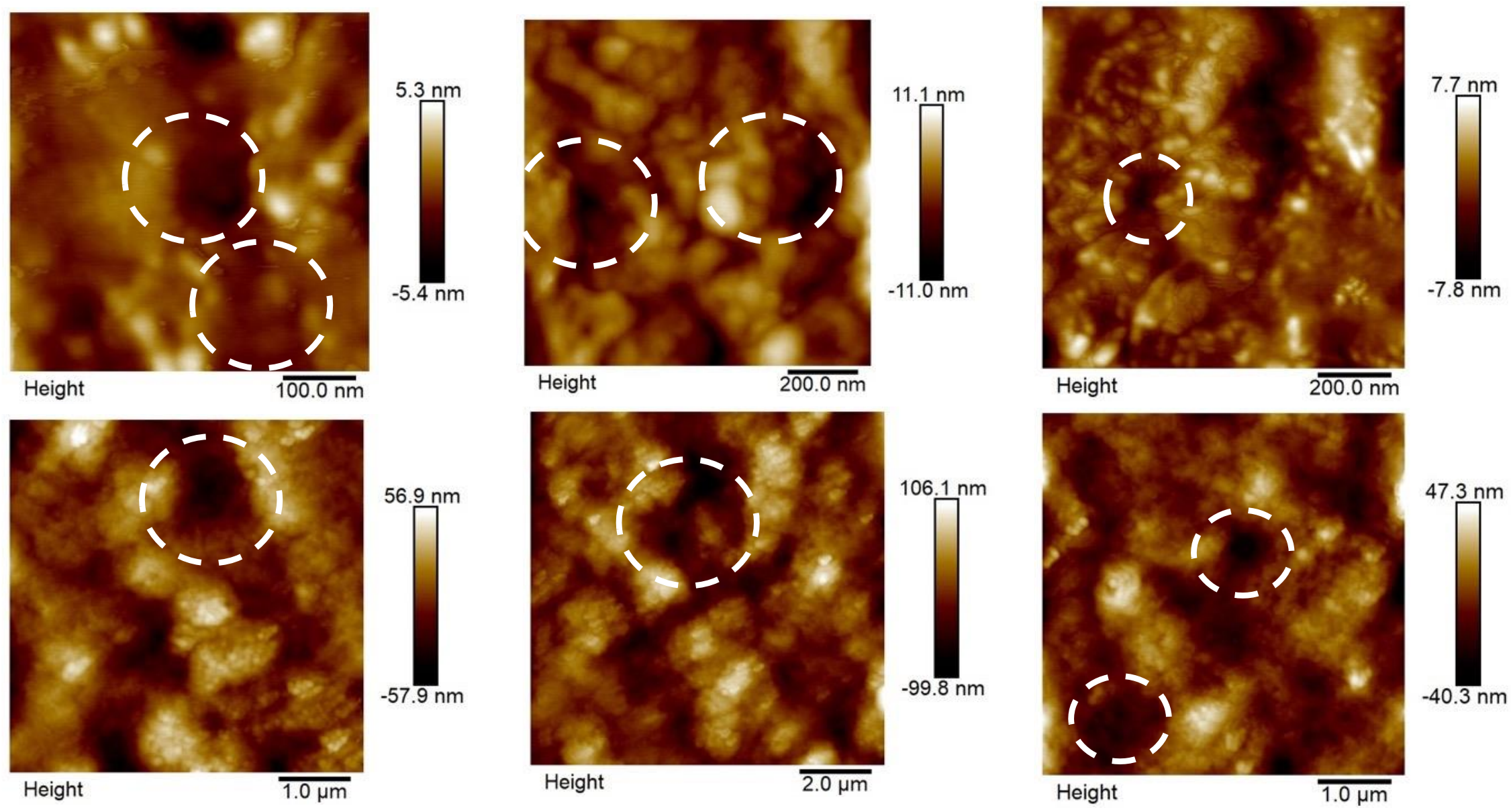

Figure S11. AFM height images of the hydrogel coating at different magnifications, used for apparent pore-size analysis.

13. **Supplementary Tables**

Table S1. Determination of carbonic anhydrase (CA) loading in nanoflowers based on residual enzyme concentration in the supernatant after synthesis

	**Abs280 ± SD**	**[CA] supernatant**	**CA loading (%)**
**CA-HNF05**	0.7960 ± 0.0159	0.4190	16.2070
**CA-HNF01**	0.1539 ± 0.0351	0.0757	24.2807
**CA-HNF005**	0.0068 ± 0.0006	0.0036	92.8070

Table S2. Comparison of catalytic parameters for bio-inspired and enzyme-based electrocatalysts to CO_2 reduction.

Catalyst	Product	Electrolyte, pH	Current density*	%FE[1]	%FE[2]	ref

NF	Formate / acetate	0.1 M Tris-HCl, 8.0	-6.19 ± 0.16 at -1.7 V	8.46 ± 0.79	7.69 ± 7.47	This work
CA-HNF05	Formate / acetate	0.1 M Tris-HCl, 8.0	-7.23 ± 0.04 at -1.7 V	9.73 ± 1.13	12.21 ± 0.80	This work
CA-HNF01	Formate / acetate	0.1 M Tris-HCl, 8.0	-8.33 ± 0.20 at -1.7 V	24.16 ± 3.35	36.24 ± 3.10	This work
CA-HNF005	Formate / acetate	0.1 M Tris-HCl, 8.0	-9.34 ± 0.12 at -1.7 V	28.94 ± 0.31	40.75 ± 2.43	This work
F/PPy-NR/DHs[a]	Methanol	0.1 M PBS, 7.4	-1.5 to -3.0 at -1.2 V	10		[8]
FDH	Formic acid	0.1 M PBS, 7.4	-6.97 at -1.0 V	12.74		[9]

* mA cm^{-2}; [1] FE to formate; [2] FE to acetate; [a]Carbon Felt/Polypyrrole/ Neutral Red/Dehydrogenases; [b] Formate dehydrogenase.

Table S3. Apparent pore diameters from AFM height images.

Pore	Scale bar	Pore diameter
**1-2**	100 nm	182-183 nm
**3-5**	200 nm	232-379 nm
**6, 8-9**	1.0 µm	1.26-1.86 µm
**7**	2.0 µm	3.71 µm

Table S4. EIS-derived parameters.

Parameter	Mean ± SD
**Rs (Ω)**	82.9 ± 0.24
**CPE**	$(3.13 \pm 0.26) \times 10^{-4}$
**Rct (Ω)**	$(6.51 \pm 6.80) \times 10^{3}$
**χ^2**	0.183 ± 0.049

Table S5. Integrated $\nu_3(CO_2)$ band area (2350 cm^{-1}) for individual regions of interest selected from the micro-FTIR hyperspectral maps of CA-HNF and NF.

CA-HNF	NF
529820	41187
517733	42942
500395	44325
478317	58481
475729	58484
471133	59686
440361	62368
418002	70420
417459	71460
412014	71649

References

(1) Lindskog, S.; Nyman, P.-O. *Amino Acid Composition of Various Forms of Bovine and Human Erythrocyte Carbonic Anhydrase*; 1964. https://doi.org/10.1016/0926-6569(64)90174-9 (accessed 2025-11-08).

(2) Albuquerque, F. F.; Iost, R. M.; Fonseca, G. C.; Venkatkarthick, R.; Pacheco, J. C.; Colombo, R. N. P.; Lima, F. H. B.; Crespilho, F. N. Biomimetic Cobalt Complex Stabilized by Hydrogel on High-Edge-Density Graphite for ORR and HER in Quiescent Solutions. *Langmuir* **2025**, *41* (34), 22738–22748. https://doi.org/10.1021/acs.langmuir.5c01683.

(3) Hoque, E.; DeRose, J. A.; Bhushan, B.; Hipps, K. W. Low Adhesion, Non-Wetting Phosphonate Self-Assembled Monolayer Films Formed on Copper Oxide Surfaces. *Ultramicroscopy* **2009**, *109* (8), 1015–1022. https://doi.org/10.1016/j.ultramic.2009.03.033.

(4) Sydorchuk, V.; Poddubnaya, O. I.; Tsyba, M. M.; Zakutevskyy, O.; Khyzhun, O.; Khalameida, S.; Puziy, A. M. Photocatalytic Degradation of Dyes Using Phosphorus-Containing Activated Carbons. *Appl. Surf. Sci.* **2021**, *535*. https://doi.org/10.1016/j.apsusc.2020.147667.

(5) Sedenho, G. C.; Nascimento, S. Q.; Zamani, M.; Crespilho, F. N.; Furst, A. L. Secondary Structure in Enzyme-Inspired Polymer Catalysts Impacts Water Oxidation Efficiency. *Advanced Science* **2024**, *11* (25). https://doi.org/10.1002/advs.202402234.

(6) Wu, X.; Gong, K.; Zhao, G.; Lou, W.; Wang, X.; Liu, W. Mechanical Synthesis of Chemically Bonded Phosphorus-Graphene Hybrid as High-Temperature Lubricating Oil Additive. *RSC Adv.* **2018**, *8* (9), 4595–4603. https://doi.org/10.1039/c7ra11691h.

(7) Sedenho, G. C.; De Porcellinis, D.; Jing, Y.; Kerr, E.; Mejia-Mendoza, L. M.; Vazquez-Mayagoitia, Á.; Aspuru-Guzik, A.; Gordon, R. G.; Crespilho, F. N.; Aziz, M. J. Effect of Molecular Structure of Quinones and Carbon Electrode Surfaces on the Interfacial Electron Transfer Process. *ACS Appl. Energy Mater.* **2020**, *3* (2), 1933–1943. https://doi.org/10.1021/acsaem.9b02357.

(8) Seelajaroen, H.; Mayr, F.; Wielend, D.; Cobet, M.; Ulbricht, C.; Serdar Sariciftci, N.; Tekoglu, S. Sustainable One-Pot Electrochemical Approach for Entrapment of Multi-Enzymes and Biocompatible Redox Mediator. *Chempluschem* **2025**, *90* (5). https://doi.org/10.1002/cplu.202400577.

(9) Srikanth, S.; Maesen, M.; Dominguez-Benetton, X.; Vanbroekhoven, K.; Pant, D. Enzymatic Electrosynthesis of Formate through CO2 Sequestration/Reduction in a Bioelectrochemical System (BES). *Bioresour. Technol.* **2014**, *165* (C), 350–354. https://doi.org/10.1016/j.biortech.2014.01.129.
